# Chloroplast phylogenomics provides new evidence for reevaluating the taxonomic placement of medicinal *Agapetes*

**DOI:** 10.3389/fpls.2025.1586413

**Published:** 2025-10-22

**Authors:** Jindong Wang, Baizhu Li, Yunjing Liu, Yu Li, Yin Yi, Wei Xie, Xiaoxin Tang

**Affiliations:** ^1^ Key Laboratory of National Forestry and Grassland Administration on Biodiversity Conservation in Karst Mountainous Areas of Southwestern China, School of Life Science, Guizhou Normal University, Guiyang, China; ^2^ Engineering Research Center of Carbon Neutrality in Karst Areas, Guizhou Normal University, Guiyang, China; ^3^ Guizhou Collaborative Innovation Center of Green Finance and Ecological Environment Protection, Guiyang, China; ^4^ School of Life Sciences, Central China Normal University, Wuhan, China; ^5^ China Agricultural University, College of Resources and Environmental Sciences, Beijing, China; ^6^ School of Chemistry, Chemical Engineering and Biotechnology, Nanyang Technological University, Singapore, Singapore

**Keywords:** *Agapetes*, phylogeny, chloroplast genome, ITS, *Vaccinium*

## Abstract

Species of *Agapetes* are recognized for their radish-like tubers, which possess significant medicinal properties. Resolving the long-standing phylogenetic controversies between *Agapetes* and its relatives is crucial for facilitating the utilization of this genus. However, the scarcity of molecular data has persistently constrained such investigations. In this study, we generated the first high-quality chloroplast (cp) genome assemblies for three pharmacologically important *Agapetes* species: *A. malipoensis*, *A. guangxiensis*, and *A. obovata*, with genome sizes of 172,729, 176,291, and 180,574 bp, respectively. Phylogenetic analyses based on both complete chloroplast genomes and nuclear internal transcribed spacer (ITS) sequences supported the monophyly of *Agapetes* and *Vaccinium*, with bootstrap values of 100% and 63%, respectively. More intriguingly, the chloroplast phylogeny placed the *Agapetes* clade nested within *Vaccinium*. Moreover, the ITS phylogeny revealed that species of *Agapetes* were intermixed with those of *Vaccinium*. This intermixed pattern was further supported by hierarchical clustering based on relative synonymous codon usage (RSCU) and the abundance of repetitive sequences, including simple sequence repeats (SSRs) and dispersed repeats. Species of the two genera exhibited no significant differences in other chloroplast genomic features, including proportions of protein-coding genes and non-coding regions, GC content across all quadripartite structural regions, IR boundary shift, and tandem repeats. These findings provide novel molecular evidence supporting the taxonomic merger of the medicinally important genera *Agapetes* and *Vaccinium*. This work establishes a critical foundation for future investigations into the evolutionary origins of medicinal traits, pharmaceutical exploration, and the precise species delimitation of *Agapetes* and *Vaccinium*.

## 1 Introduction

*Agapetes* (Ericaceae) is primarily distributed from the eastern Himalayas to Southeast Asia. This genus comprises approximately 115 species, with 63 of them native to China (Tong et al., 2024; POWO, 2025; Zou et al., 2025). Species of *Agapetes* are primarily epiphytic, growing on tree trunks in dense forests or lithophytic on rocky outcrops in open shrublands (Conlon, 2015). They are highly prized in horticulture for their pendulous, bell-shaped flowers that exhibit vibrant colors (Conlon, 2015). *Agapetes* are also known for their radish-like tubers, which contain a diverse range of secondary metabolites, such as phenols, tannins, polysaccharides, saponins, flavonoids, lactones, coumarins, organic acids, and sterols (Yan et al., 2019). These metabolites are useful for dispersing blood stasis, relieving pain, promoting diuresis, reducing swelling, and providing anti-inflammatory effects (Chen et al., 1990). Tubers of certain *Agapetes* species are traditionally used to enhance lactation and support postpartum recovery in nursing mothers (Jariya et al., 2011). For the further development and utilization of *Agapetes* species, it is essential to clarify the phylogenetic position of this genus.

The taxonomic delineation between *Agapetes* and its close relative *Vaccinium* has long been debated. In *Agapetes*, the corolla is usually elongated, tubular, narrowly funnel-shaped, or campanulate; stamens are slightly adherent and encircling the style or free; pedicels are often thickened toward the apex, sometimes becoming cup-shaped; plants are usually epiphytic, rarely terrestrial (Stevens, 1972, 1985; Vander Kloet, 1988; Fang et al., 2005). In *Vaccinium*, the corolla is relatively short, typically urceolate or campanulate, rarely tubular; stamens are free and do not encircle the style; the apex of the pedicel is generally not thickened; plants are usually terrestrial, occasionally epiphytic (Stevens, 1972, 1985; Vander Kloet, 1988; Fang et al., 2005). Some researchers have argued for merging the two genera into *Vaccinium* based on their morphological similarities (Stevens, 1985; Kron et al., 2002; Vander Kloet, 2004). Phylogenetic results based on internal transcribed spacer (ITS) and *matK* gene sequence indicated that *Agapetes* are closely related to *Vaccinium* (Kron et al., 2002). The similarity of *rolB/C*-like gene sequences between *Vaccinium* and *Agapetes* suggests that they may share a common origin (Zhidkin et al., 2023). All these investigations indicate the difficulty in separating *Agapetes* and *Vaccinium* as different genera. Stronger molecular evidence is required, given the low resolution of existing phylogenetic signals.

Most chloroplast (cp) genomes are characterized by a typically circular quadripartite structure consisting of one large single copy (LSC), one small single copy (SSC), and two copies of the inverted repeat (IR) region, which look like LSC-IRb-SSC-IRa-LSC (Palmer, 1983; Daniell et al., 2016). Sequences at the ends of the IR regions are regarded as IR boundaries (Daniell et al., 2016). The mutation rate of the cp genome is moderate, approximately one-third that of nuclear genes and three times that of mitochondrial genes (Drouin et al., 2008). These properties made cp genomes an ideal tool for plant phylogenetic study and species identification (Wang et al., 2024). Moreover, such applications have been widely implemented in plants, including an increasing number of medicinal plants, driven mainly by improvements in sequencing technology, assembly methods, and bioinformatic tools (Wu and Ge, 2012; Wang et al., 2024).

Phylogenetic investigation based on the cp genome revealed the closer relationship between *Houttuynia cordata* and *Aristolochia*, laying the groundwork for further studies of these taxa (Zhu et al., 2020). The analysis of the cp genomes in *Carpesium* and *Atractylodes* illuminates the interspecific relationships and evolutionary history of these medicinal plants, providing new molecular markers for species identification and genetic diversity research (Wang et al., 2021; Shi et al., 2022). Cp genomes based on research on the evolutionary relationship between *Paeonia ostii* and relatives offer potential chances for enhancing peony yield (Guo et al., 2018). These studies not only enrich the available data on cp genomes of medicinal plants but also offer novel avenues for genetic improvement and breeding of these valuable species.

Overall, *Agapetes* species possess important medicinal attributes. Clarifying the phylogenetic circumscription of *Agapetes* is a prerequisite for accurately identifying species within this genus and the utilization of its pharmaceutical potential. DNA sequence, especially the cp genome, is effective data for the construction of phylogenies. Existing research about phylogenetic relationships between *Agapetes* and allied taxa predominantly relied on morphological characteristics or short DNA sequence fragments (e.g., chloroplast gene *matK*) (Camp, 1940; Vander Kloet, 1988, 2004). In this study, we investigated the phylogenetic position of *Agapetes* using complete cp genome sequences. Concurrently, we obtained extensive ITS sequence data from *Agapetes* and related taxa, particularly *Vaccinium*, to conduct large-scale phylogenetic analyses and comparative assessments. This integrated research provides robust evidence for resolving the systematic evaluation of *Agapetes*.

## 2 Materials and methods

### 2.1 Plant materials

All three *Agapetes* species, namely, *A. malipoensis*, *A. guangxiensis*, and *A. obovata* used in this work, are native to Malipo County, Yunnan Province, China (**Supplementary Figures S1, S2**). Before sampling, based on morphological characteristics, the three species were identified by taxonomist Chao Zhang from the School of Life Sciences, Guizhou Normal University, and Su Zhang from the School of Forestry, Beijing Forestry University (**Supplementary Figure S2**). Fresh leaves were collected from one individual of *A. malipoensis* (23.0496°N, 104.8130°E), *A. guangxiensis* (23.1837°N, 104.8279°E), and *A. obovata* (23.0449°N, 104.8209°E), respectively. The sampled leaves were rinsed with sterile water, dried with absorbent paper, and then put into tea bags, which were sealed inside plastic bags together with moisture-absorbing silica gel and appropriately labeled for long-term storage and DNA extraction. One voucher specimen for each of the three species was deposited in the Herbarium of Guizhou Normal University with special voucher numbers: GZUB-20240925–0001 for *A. malipoensis*, GZUB-20240925–0002 for *A. guangxiensis*, and GZUB-20240925–0003 for *A. obovata*.

### 2.2 DNA extraction and sequencing

Total DNA was extracted from the leaves of the three *Agapetes* species using the modified cetyltrimethylammonium bromide (CTAB) method (Doyle and Doyle, 1987). The DNA was then fragmented using ultrasonication, followed by purification, end-repair, 3′ adenylation, and ligation of sequencing adapters. Finally, after fragment size selection using agarose gel electrophoresis, PCR amplifications were performed to generate the sequencing library. Illumina NovaSeq 6000 was used for final sequencing with paired-end (PE) mode, generating raw reads of 150 bp. The raw reads were filtered using fastp v0.23.4, and adapters, primers, and reads whose average score was lower than Q5 or ambiguous bases (denoted as “N”) more than five were dropped (Chen, 2023).

### 2.3 Assembling, annotation, and assessment of chloroplast genomes

Filtered reads of *A. malipoensis* were first assembled using NOVOPlasty v4.2 (Dierckxsens et al., 2017). Then, the assembled contigs were further aligned to the NT database of NCBI, so that the contigs from the cp genome were identified. Filtered reads of *A. guangxiensis* and *A. obovata* were mapped to published cp genomes with bowtie2 v2.2.4 in very-sensitive-local mode to identify cp genome derivation (Langmead and Salzberg, 2012). SPAdes v3.10.1 was used to generate the primary contigs based on k-mers, including 55-mer, 87-mer, and 121-mer (Bankevich et al., 2012). For the three species, SSPACE v2.0 was applied for cp genome scaffolding based on contigs (Boetzer et al., 2011). The gaps in the cp genomes were then filled using Gapfiller v2.1.1 (Boetzer et al., 2012).

Online platform cpGAVAS2 was employed for the predictions of protein-coding genes (PCGs), rRNA genes, and tRNA genes of the three cp genomes (Shi et al., 2019). Their quadripartite structures were identified based on homologous alignments to their relatives using MUMmer v3.1 (**Supplementary Table S1**) (Marçais et al., 2018). To assess the results of assembling and annotating, the online tool PMGmap was used to visualize the three cp genome circular maps (Zhang et al., 2024). Finally, the three assemblies were submitted to NCBI with specific accession numbers (**Supplementary Table S1**).

### 2.4 Data sources and phylogenetic analyses

The three cp assemblies of *Agapetes* and high-quality cp genomes of their 12 relatives downloaded from NCBI GenBank were used for further investigations (**Supplementary Table S1**). Relative synonymous codon usage (RSCU) values were calculated for all protein-coding genes across the chloroplast genomes using CodonW v1.4.2 (https://codonw.sourceforge.net/). The resultant RSCU matrix was subjected to hierarchical clustering and visualized through a heatmap generated with the online platform ChiPlot (https://www.chiplot.online/).

For chloroplast phylogenomic analyses, the PCG sequences of these cp genomes were first extracted using PhyloSuite v1.2.3 based on annotation results (Xiang et al., 2023). Single-copy genes of these species were then concatenated as one supergene alignment, after multiple sequence alignment (MSA) implemented with MAFFT v7.475 and further refined with Gblocks v0.9b (Castresana, 2000; Katoh and Standley, 2013). ModelFinder v1.6.12 was employed to find the best substitution models for chloroplast phylogenomic reconstruction based on Bayesian inference (BI) and maximum-likelihood (ML) frameworks (Kalyaanamoorthy et al., 2017). BI-based chloroplast phylogenomic relationships were reconstructed by MrBayes v3.2.7 with the GTR+F+I+G4 substitution model (Ronquist et al., 2012), and two independent runs were employed for convergent results. For each run, two million generations were performed with sampling every 1,000 generations, and the first 25% were discarded as burn-in. The ML reconstructions were implemented using IQ-tree v2.2.2.7 with the GTR+F+R2 substitution model and 5,000 bootstrap replicates (Nguyen et al., 2015). *Solanum melongena* was used as an outgroup in both BI and ML chloroplast phylogenomic analyses.

The ribosomal DNA sequences of the nuclear genomes of *A. malipoensis*, *A. guangxiensis*, and *A. obovata* were assembled from their filter reads mentioned above using GetOrganelle v1.75 (Jin et al., 2020). Subsequently, their ITS sequences, including ITS1, 5.8S ribosomal DNA, and ITS2, were extracted using ITSx v1.1.3 (Bengtsson-Palme et al., 2013). ITS sequences of related species were downloaded from NCBI GenBank (**Supplementary Table S2**). The same methods were used in ITS, ITS1, ITS2, and chloroplast genome phylogenetic reconstructions, but the best substitution model was TNe+G4 for BI and TN+F+G4 for ML estimation, and the outgroup was *Gaultheria griffithiana*. Multisequence alignment of ITS was visualized by Jalview v.2.11.4.0 (Waterhouse et al., 2009). Phylogenetic trees were visualized with tvBOT (https://www.chiplot.online/tvbot.html).

### 2.5 Analyses of repetitive sequences

Simple sequence repeats (SSRs) in cp genomes of *Agapetes* species and their relatives were identified by the online tool Misa with the following search parameters (motif length—min. no. of repetitions): 1—10 (mononucleotide), 2—5 (dinucleotide), 3—4 (trinucleotide), 4—3 (tetranucleotide), 5—3 (pentanucleotide), and 6—3 (hexanucleotide) (Beier et al., 2017). Dispersed repeats, including forward, reverse, complemented, palindromic, and reverse-complemented repeats of these species, were reported by REPuter online version, and the parameters, including hamming distance, minimal repeat size, and maximum computed repeats, were set as 3, 30, and 5,000 (Kurtz, 2001). Tandem repeats were detected using the online tool TRF (Benson, 1999). Visualization of the results was performed in R v4.3.2 using the ggplot2 package (Wickham, 2016). Multisequence alignment of repeats was visualized by the R package ggmsa (Zhou et al., 2022). Finally, CPJdraw v1.0.0 was used to analyze and visualize the IR region boundaries of these cp genomes (Li et al., 2023). The online platform ChiPlot (https://www.chiplot.online/) was used to visualize the counts of SSRs and dispersed repeats.

### 2.6 Chloroplast genomic comparisons

The online platform mVISTA was used to calculate and show variations of cp genomes between *Agapetes* species and relatives with the Shuffle-LAGAN alignment model, and *Agapetes malipoensis* was set as the reference (Brudno et al., 2003; Frazer et al., 2004). For each cp genome, the GC content of different regions, protein-coding gene content, and proportions of coding and non-coding regions were calculated by PhyloSuite v1.2.3 (Xiang et al., 2023). The cp genomes were also divided into three groups by taxa: AGA (*Agapetes*), VAC (*Vaccinium*), and OUT (other taxa). Then, the generalized linear model (GLM) in IBM SPSS Statistics 26 was applied for between-group variation detection of GC content, gene content, etc.

## 3 Results

### 3.1 Cp genomic characteristics of the three *Agapetes* species

**Figure 1:**
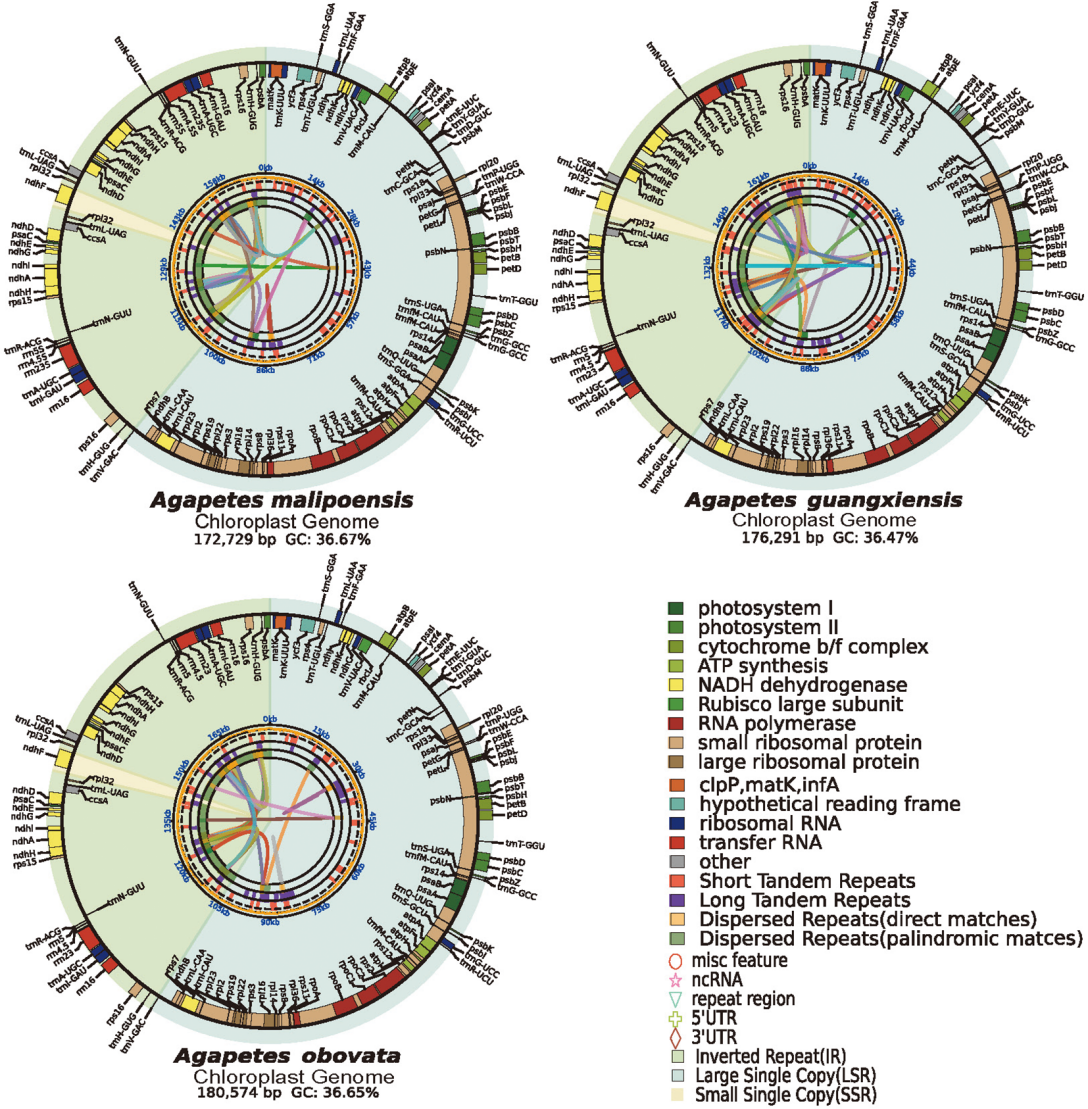
Chloroplast genome maps of *Agapetes* species. The chloroplast genome map comprises four concentric tracks radiating, which show the relationships between dispersed repeats, dispersed repeats, tandem repeats, microsatellite distribution (SSRs), and colored functional gene map from the center outward. The quadripartite structure is demarcated by shaded sectors, yellow for SSC, green for LSC, and chartreuse for IRa and IRb regions.

For *A. malipoensis*, *A. guangxiensis*, and *A. obovate*, 5.67, 12.49, and 10.37 Gb of pair-end short reads were generated, respectively (**Supplementary Table S3**). Their assembled cp genomes were characterized by conserved quadripartite structures (LSC, SSC, and two IR regions), with lengths of 172,729, 176,291, and 180,574 bp and GC content of 36.67%, 36.47%, and 36.65% (**Figure 1**; **Supplementary Table S4**). Annotations of the three cp genomes were highly consistent. In *A. malipoensis*, 130 genes, consisting of 85 protein-coding genes, 37 tRNA genes, and 8 rRNA genes, were predicted. According to functions and structures, these genes were divided into four categories: photosynthesis-related genes, self-replication-related genes, other genes, and genes of unknown function (**Supplementary Tables S5–7**). Photosynthesis-related genes were classified into six groups, namely, subunits of photosystems I and II, NADH dehydrogenase, cytochrome b/f complex, ATP synthase, and the large subunit of Rubisco. Self-replication-related genes consist of large/small ribosomal subunit proteins, subunits of RNA polymerase, ribosomal RNAs, and transfer RNAs. The two conserved hypothetical chloroplast ORFs, *ycf3* and *ycf4*, were viewed as genes of unknown function. *Matk*, *cermA*, and two copies of *ccsA* were contained in other genes (**Supplementary Tables S5–7**). We identified 22 genes that have undergone duplication, including *psaC*, *psbA*, *trnR-ACG*, *ccsA*, and others. Specifically, 11 of these duplicated genes are protein-coding genes, 4 are rRNA genes, and 7 are tRNA genes (**Supplementary Tables S5–7**).

### 3.2 Phylogenetic status of *Agapetes*

**Figure 2:**
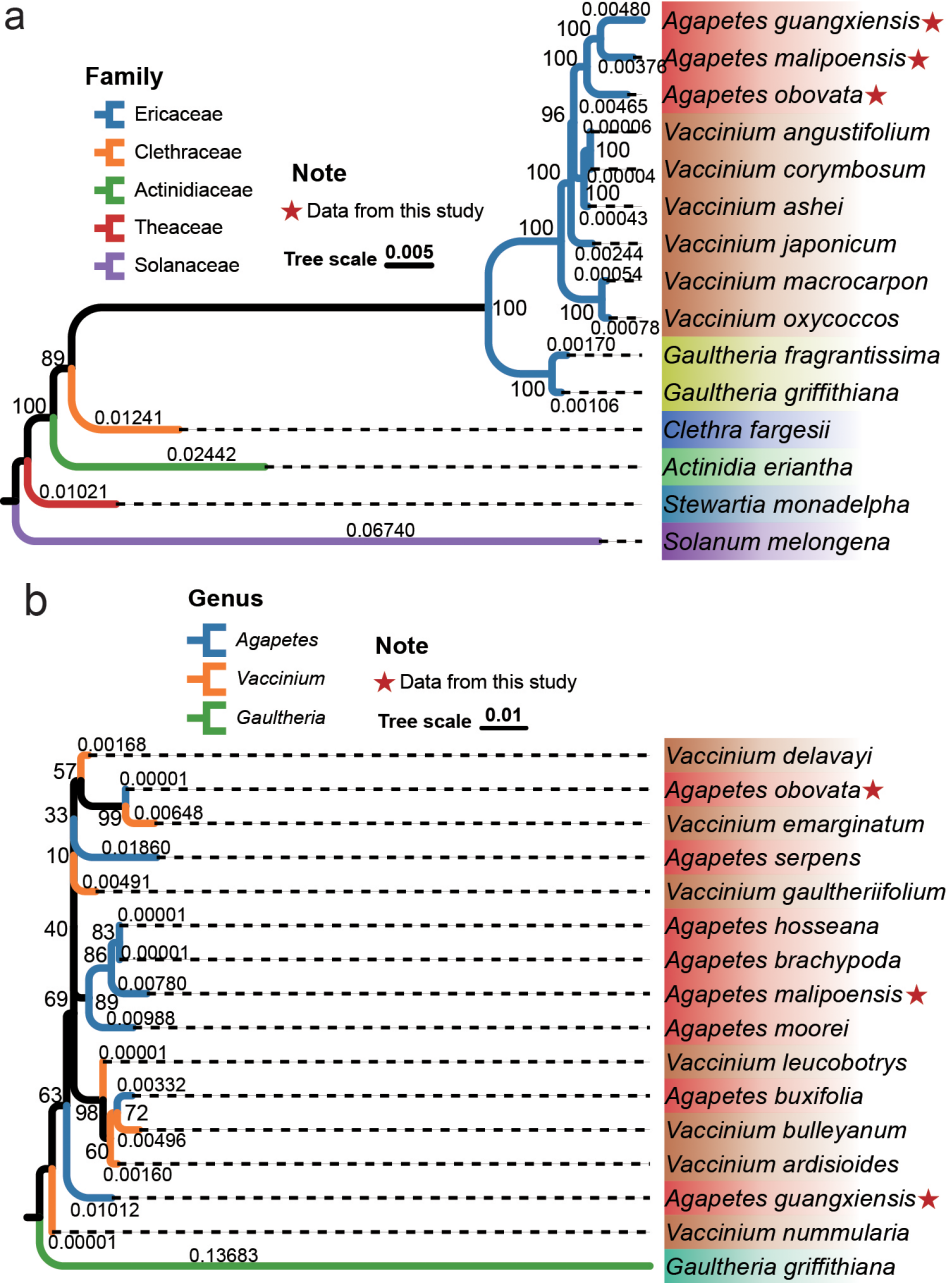
Phylogenetic relationships of *Agapetes* and closely related species. ML phylogeny of chloroplast genomes **(a)** and ITSs **(b)**; bootstrap values are shown beside each node, and branch lengths are shown above the clades.

Chloroplast genomes of the three *Agapetes* species and their 12 relatives from five families were used to reconstruct an ML phylogeny, which was generally consistent with the APG IV classification system (**Figure 2a**). The ML phylogeny was supported by bootstrap values larger than 89% for all internal nodes (**Figure 2a**). *Solanum melongena* of Solanaceae was set as an outgroup. Species from Theaceae, Actinidiaceae, Clethraceae, and Ericaceae formed four distinct clades. Two *Gaultheria* species of Ericaceae formed a single clade. The three *Agapetes* species formed a clade nested in *Vaccinium*, with a significantly larger genetic distance to *V. japonicum*, *V. macrocarpon*, and *V. oxycoccos* than to other species within *Vaccinium* (**Figure 2a**). This relationship was also supported by BI (**Supplementary Figure S4a**).

ITS sequences are nuclear genome-derived DNA commonly used for phylogenetic investigations. The ITS region comprises ITS1, 5.8S rDNA, and ITS2. ITS sequences from more species of *Agapetes* and *Vaccinium* were used for further validation of their relationship based on ML and BI methods (**Figures 2b**, **S3, S4b**). Sequences of *Gaultheria griffithiana* were used as an outgroup. The ITS phylogeny also supported the monophyly of the two genera. However, the species were intermixed and did not form two distinct groups corresponding to the current circumscription of *Agapetes* and *Vaccinium* (**Figures 2b**, **S4b**). Similar phylogenetic relationships were found from ITS1 and ITS2 based on ML and BI methods (**Supplementary Figure S5**). This result was also supported by hierarchical clustering based on the RSCU (**Supplementary Figure S6**; **Supplementary Table S8**).

Morphological similarities also provided additional support to the DNA sequence phylogenies. The flowers of *A. guangxiensis* were similar to those of *V. vitis-idaea* (**Supplementary Figure S2**). The fruits of *A. obovata* were similar to those of *V. vitis-idaea*, *V. henryi*, and *V. dunnianum* (**Supplementary Figure S2**). Most species of *Agapetes* and *Vaccinium* possessed five triangular calyx lobes with an outer surface often glabrous (**Supplementary Tables S9, S10**). Their corollas were usually tubular or urceolate (**Supplementary Tables S9, S10**). Similarities in other parts were also recorded, including the style, filament, fruit, leaf, and phenology (**Supplementary Tables S9, S10**).

### 3.3 Low cp genome variation between *Agapetes* and *Vaccinium*

**Figure 3:**
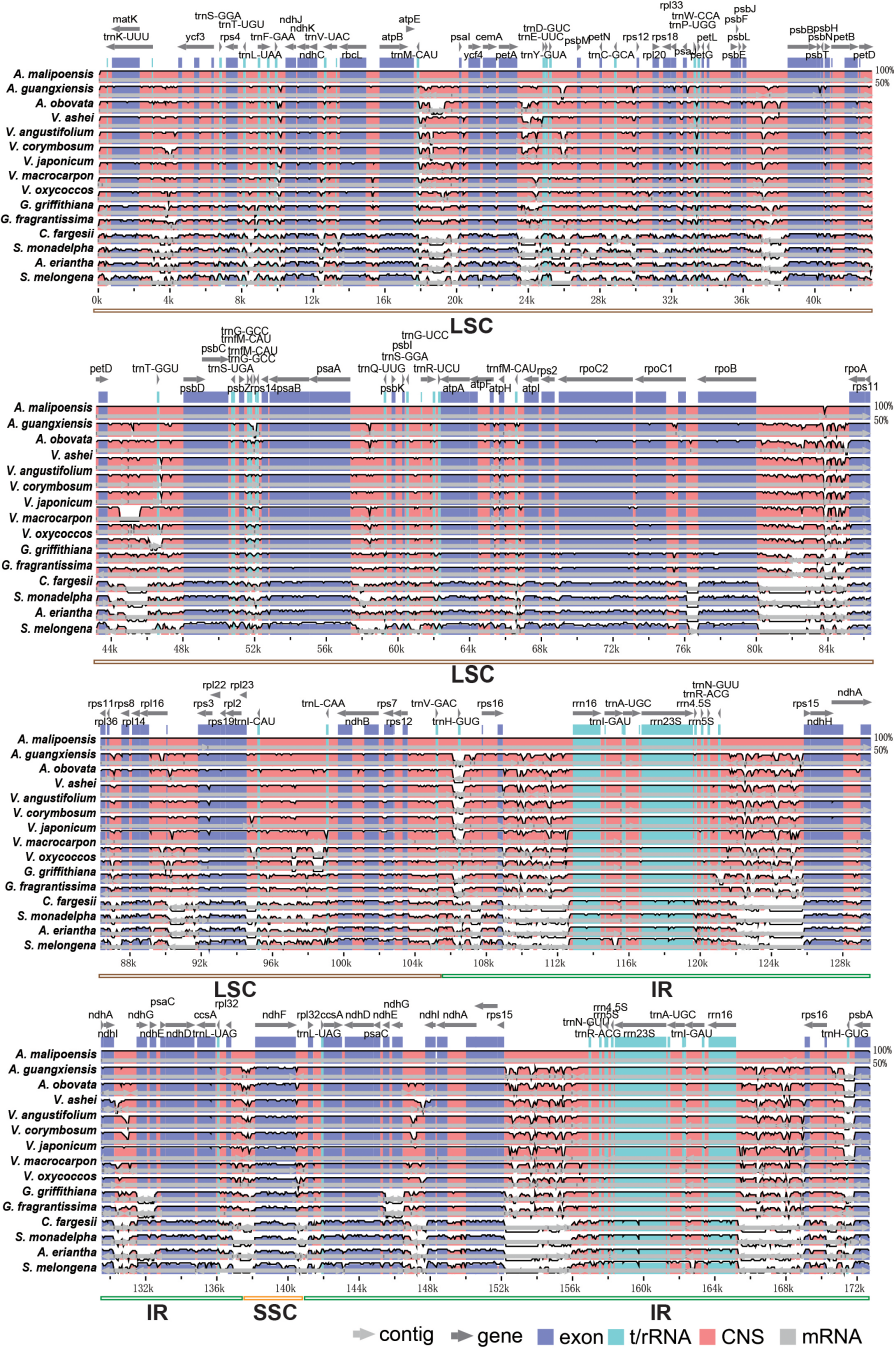
Whole chloroplast genome alignment of *Agapetes* and relatives. *A. malipoensis* was used as reference. The top line shows genes in order (transcriptional direction indicated by arrows). The *y*-axis represents the identity between 50% and 100%. The *x*-axis represents the coordinate.

Chloroplast genomes of the three *Agapetes* species and their 12 relatives were compared, with *A. malipoensis* as the reference (**Figure 3**). The similarity between *Agapetes* and *Vaccinium* was much more than that between *Agapetes* and other taxa, including protein-coding regions, especially the genes *ndhG*, *ndhE*, *ndhF*, *atpF*, *atpH*, *atpl*, *rps2*, and *rpoC1*, and the IR region (**Figure 3**). There were more conserved non-coding sequences for *Agapetes* and *Vaccinium* than for *Agapetes* to other species. Species in *Agapetes* and *Vaccinium* shared almost identical coding sequences except for the genes *rps3* and *ndhF* (**Figure 3**). Their variations were mainly found in non-coding regions, including the IR region and intergenic regions like *trnM-CAU*-*psaI*, *petA*-*trnE-UUC*, *rpoB-ropA*, *trnV-GAC*-*rpl23*, *rps16*-*rrn16*, and *rps3*-*rps15* (**Figure 3**).

### 3.4 Cp genomic composition similarity of *Agapetes* and *Vaccinium* species

**Figure 4:**
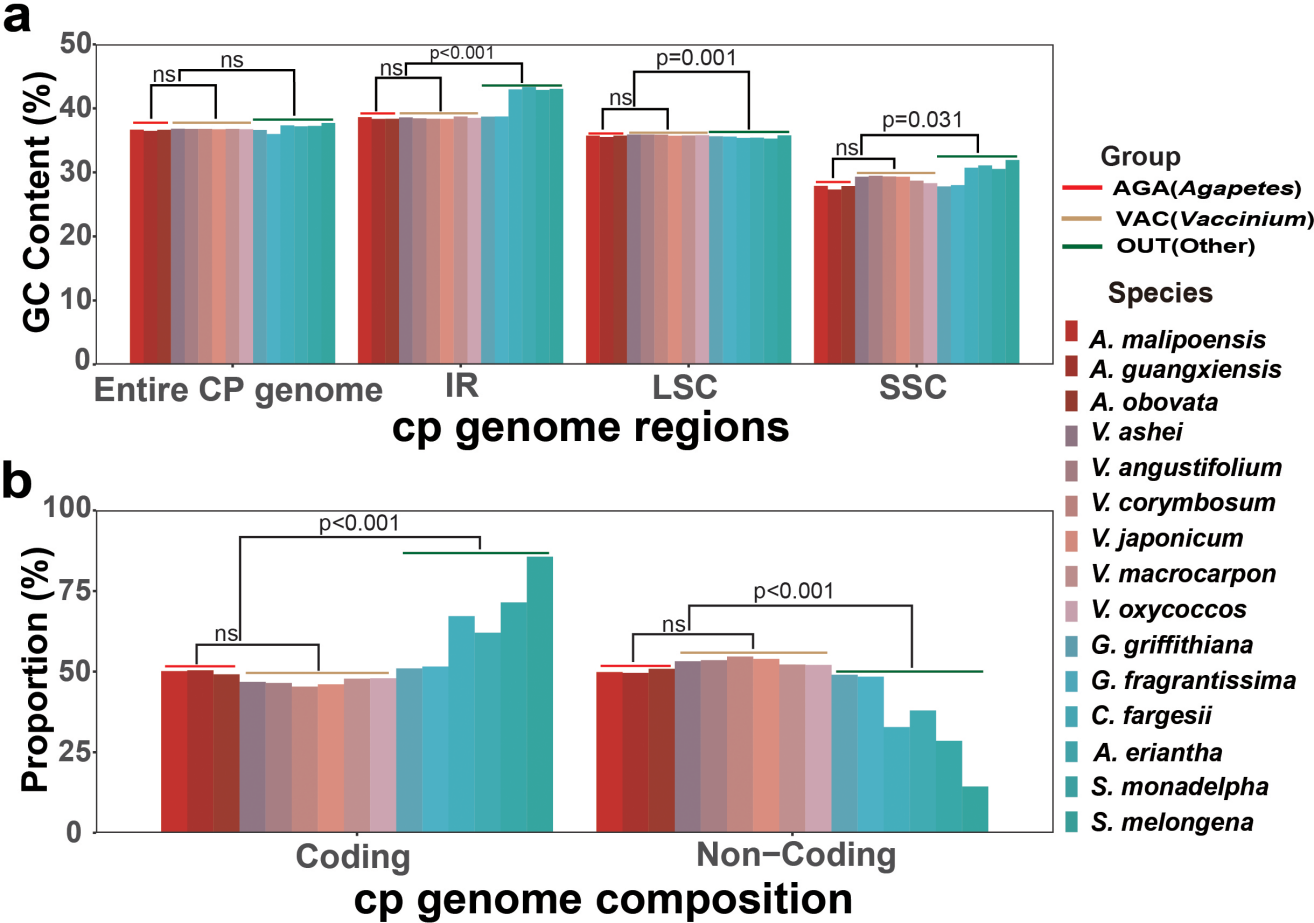
Chloroplast genome composition. **(a)** GC content of different quadripartite regions. **(b)** Proportion of coding and non-coding sequences to the whole chloroplast genome.

According to taxonomy, the 15 cp genomes were categorized into groups AGA (*Agapetes*), VAC (*Vaccinium*), and OUT (other taxa) (**Figure 4a**). The GC content of these cp genomes ranged from 36.47% to 37.71% (**Figure 4A**; **Supplementary Table S11**). The overall GC content of the entire chloroplast genome showed no significant differences in pairwise comparisons among the three groups (**Figure 4a**; **Supplementary Table S11**). The GC content of the SSC region (27.32%~31.94%) was the lowest compared to other regions (35.52%~43.05%) for all species (**Figure 4a**; **Supplementary Table S11**). In the SSC, LSC, and IR regions, the GC content difference was not significant between groups AGA and VAC (*p* > 0.05), but significant (*p* ≤ 0.001 or *p* < 0.05) between them and group OUT (**Figure 4a**; **Supplementary Table S11**). A similar pattern was detected for gene number, proportion of coding sequences, and non-coding sequences of the cp genomes of the three groups (*p* ≤ 0.001) (**Figure 4b**; **Supplementary Table S12**).

### 3.5 Repeat sequence characteristics of *Agapetes* and *Vaccinium* species

**Figure 5:**
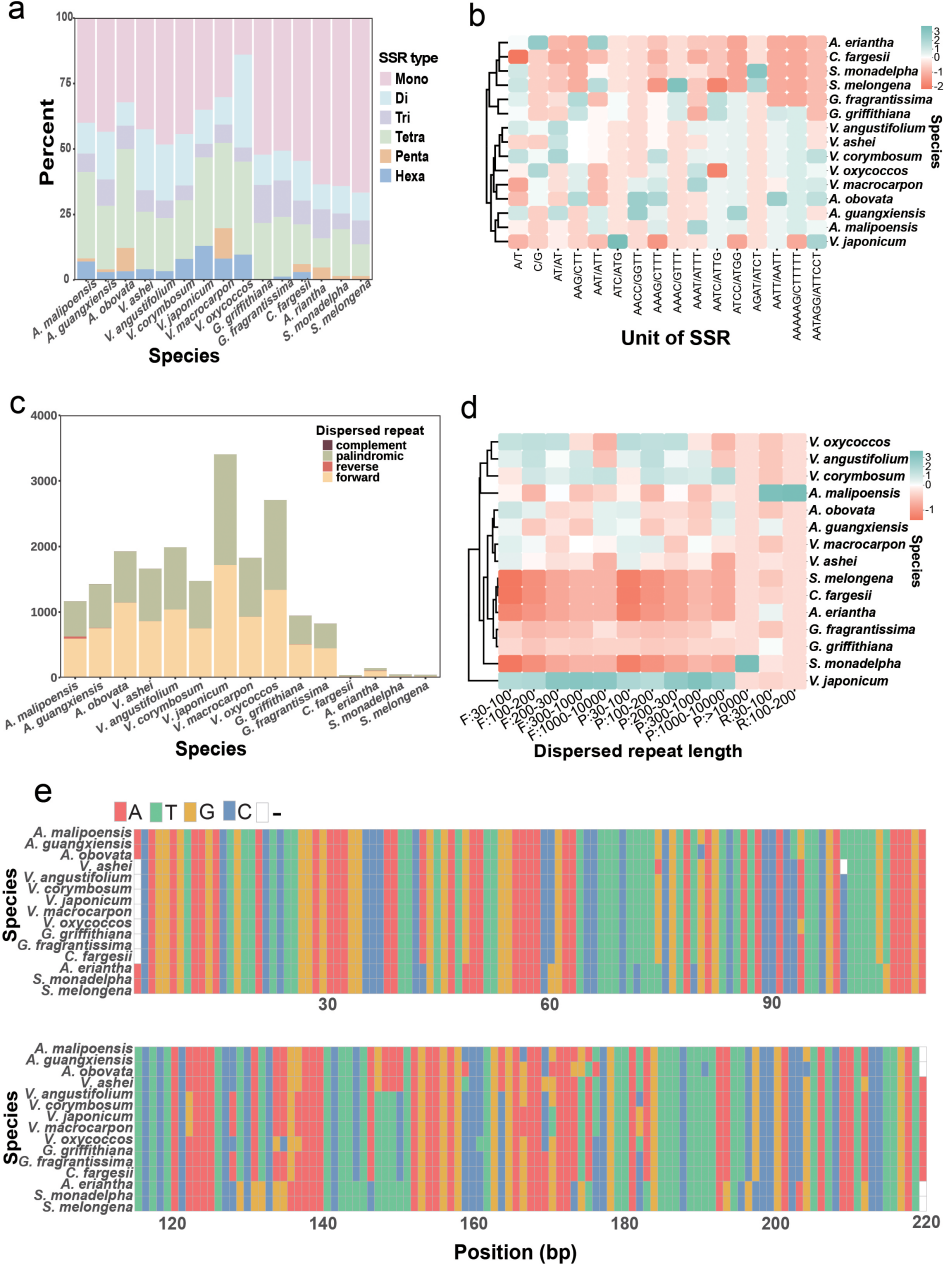
Repeat sequence characteristics of chloroplast genomes of *Agapetes* and relatives. **(a)** Proportional distribution of six SSR types: mono- to hexa-nucleotide repeats across taxa. **(b)** SSR unit abundance heatmap: hierarchical clustering of motif frequencies, color-scaled from low (red) to high (cyan). **(c)** Dispersed repeat classification: counts of forward, palindromic, reverse, and complement repeats. **(d)** Length-dependent repeat distribution: heatmap categorizing repeats into different intervals. **(e)** Multiple sequence alignment of the 219-bp tandem repeat consensus sequences.

Comparative analyses revealed striking differences in SSR distribution patterns. Chloroplast genomes of *Agapetes* and *Vaccinium* species harbored significantly higher SSR abundance (73–107 loci; *V. ashei* to *V. corymbosum*) than other taxa (33–73 loci; *C. fargesii* to *G. griffithiana*) (**Supplementary Table S13**). While mononucleotide SSRs dominated across all species (predominantly A/T repeats) (**Supplementary Table S13**), their relative contribution to total SSR content was notably lower in *Agapetes*/*Vaccinium* compared to outgroups (**Figure 5a**). Notably, *A. obovata* uniquely shared G/C mononucleotide SSRs with four *Vaccinium* species (*V. angustifolium*, *V. corymbosum*, *V. macrocarpon*, *V. oxycoccos*) (**Supplementary Table S13**). Furthermore, hexanucleotide SSRs showed marked taxonomic divergence: *Agapetes* and *Vaccinium* contained 3–11 such loci, contrasting sharply with only one in *G. griffithiana* and *C. fargesii*, and complete absence in four other relatives (**Supplementary Table S13**).

The chloroplast genomes of *Agapetes* and *Vaccinium* species exhibited significantly higher numbers of dispersed repeat elements (1,163–3,405 repeats) compared to their Ericaceae relatives (**Figure 5c**; **Supplementary Table S14**). Specifically, the congeneric species *G. griffithiana* and *G. fragrantissima* contained 943 and 822 repeats, respectively (**Figure 5c**; **Supplementary Table S14**). In contrast, only 37–144 dispersed repeats were identified in chloroplast genomes of phylogenetically distant taxa (**Figure 5c**; **Supplementary Table S14**). Notably, palindromic repeats and forward repeats dominated in all species, and reverse and complement repeat elements were not detected in the majority of analyzed species (**Figures 5c**, **S7** **;** **Supplementary Table S14**). Hierarchical clustering based on SSRs and dispersed repeat abundance also supported the intermixed phylogeny of *Agapetes* with *Vaccinium* species (**Figures 5b, d**).

Most tandem repeats across all species were short motifs (<50 bp) (**Supplementary Figure S8**). Interestingly, a 219-bp tandem repeat motif was identified exclusively in Ericaceae, including species in *Agapetes*, *Vaccinium*, and *Gaultheria* (**Supplementary Figure S8**; **Supplementary Table S15**). Strikingly, consensus sequence alignment of the 219-bp tandem repeats revealed minimal divergence between *Agapetes* and *Vaccinium* (4 fixed nucleotide substitutions), contrasting sharply with their divergence from *Gaultheria* (22 substitutions) (**Figure 5e**).

### 3.6 Conservation of IR region boundaries

**Figure 6:**
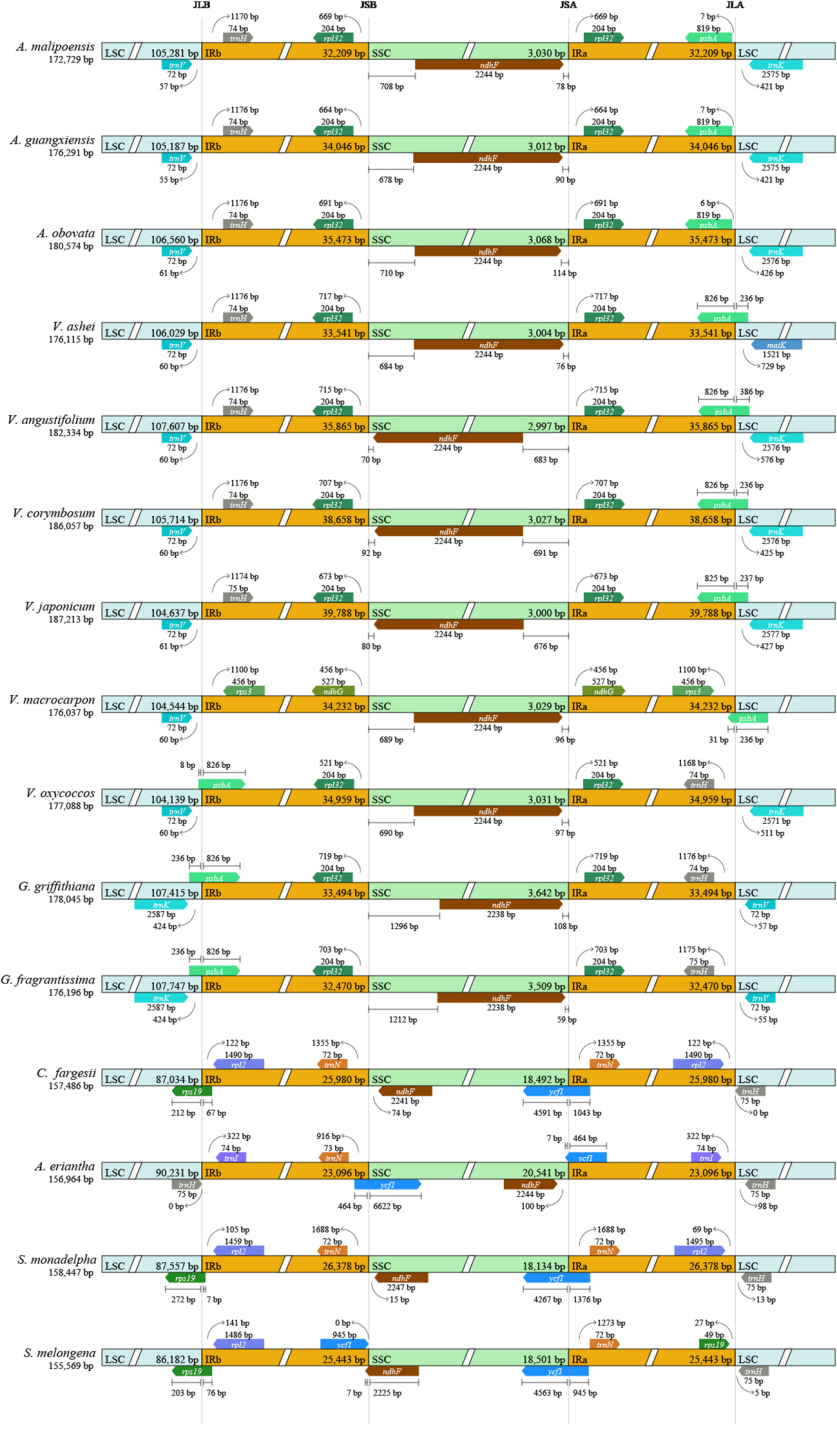
IR boundary dynamics in *Agapetes* and relatives. Annotated boxes above and below the central axis indicate flanking genes. Only structural variations at or near the IR-LSC/IR-SSC junctions are depicted.

Compared to other taxa, most species in *Agapetes* and *Vaccinium* possessed characteristics like LSC regions from 104 to 107 kb, IR regions longer than 32 kb, and SSC regions less than 3.1 kb in length (**Figure 6**). In *Agapetes*, *Vaccinium*, and *Gaultheria*, IR-LSC junctions were consistently flanked by *trnH*, *psbA*, *trnV*, and *trnK*, while IR-SSC boundaries contained *rpl32* and *ndhF*. Notably, almost all species of *Vaccinium* and *Agapetes* shared identical gene and gene order at IR boundaries, contrasting with *Gaultheria* and distantly related taxa (**Figure 6**). Overall, the IR region boundaries showed no significant expansion or contraction between *Agapetes* and *Vaccinium*, whereas considerable variation was observed in other taxa (**Figure 6**). It is therefore difficult to distinguish species of *Agapetes* and *Vaccinium* based on the lengths of the LSC and SSC regions or shifts in IR boundaries.

## 4 Discussion

Species of *Agapetes* and *Vaccinium* possess significant medicinal and nutritional value. The two genera exhibit numerous shared morphological features (**Supplementary Figure S2**; **Supplementary Tables S9, S10**). For example, both conform to the typical floral structure of Ericaceae, characterized by a 5-lobed calyx, a 5-lobed corolla, 10 stamens, and a single style. They possess leathery leaf blades arranged alternately or nearly verticillate, corollas that are usually tubular or campanulate, and a calyx tube adnate to the ovary. Their phenology is also similar, typically flowering in spring to summer and fruiting in autumn to winter. The distinction between the two genera mainly relies on the morphology of the pedicel and calyx tube, with leaf venation providing supplementary characters (**Supplementary Figure S2**; **Supplementary Tables S9, S10**). For example, in terms of pedicel morphology, *Agapetes* typically has a pedicel that expands into a club-like shape and is glabrous and longer, while *Vaccinium* has a pedicel that does not thicken and is shorter and occasionally pubescent. Regarding the calyx, the calyx in *Agapetes* is larger and variable in shape, whereas the calyx in *Vaccinium* is smaller and mostly bell-shaped or tubular. Additionally, in terms of leaf characteristics, *Agapetes* leaves are mostly oblong or lanceolate, with secondary veins and fine veinlets often prominently raised and frequently anastomosing on the adaxial surface. In contrast, *Vaccinium* leaves are mostly orbicular or ovate, with highly variable venation (Fang et al., 2005) (**Supplementary Figure S2**; **Supplementary Tables S9, S10**). Because there appears to be intergradation between these characters (Stevens, 2004), no unified morphological criterion for *Agapetes* and *Vaccinium* has been established to date.

As a consequence, classifications based solely on traditional morphology and short DNA markers (e.g., *matK*) have remained controversial, and the taxonomic boundaries as well as intergeneric relationships between *Vaccinium* and *Agapetes* are still unresolved (Tsai et al., 2003; Hummer et al., 2017; Becker et al., 2024). The plant cp genome is characterized by moderate evolutionary rates and is cost-effective for sequencing and assembling (Ané et al., 2005; Wang et al., 2024). Furthermore, chloroplast genomes possess abundant phylogenetic information, making them powerful tools for addressing taxonomic controversies, particularly among closely related species that are difficult to distinguish with conventional markers (Li et al., 2022; Xia et al., 2022; Wang et al., 2023, 2024; Kan et al., 2024; Liang et al., 2025). Nevertheless, *Agapetes* remains critically deficient in plastome data and related investigations, which impedes both the exploitation of its medicinal potential and the resolution of its taxonomic placement.

This study sequenced and assembled chloroplast genomes from three *Agapetes* species (**Figure 1**; **Supplementary Tables S1, S3-S7**). Combined with publicly available data from allied taxa (including *Vaccinium*), we reconstructed Ericaceae phylogenies using both ML and BI frameworks (**Figures 2a**, **S4a**). The resulting trees exhibited topological congruence between ML and BI phylogenies with maximum statistical support at all internal nodes (ML bootstrap = 100%; BI posterior probability = 1) (**Figures 2a**, **S4a**). The phylogenies were also consistent with the APG IV classification system (Byng et al., 2016). These convergent results robustly support the reliability of our chloroplast phylogenomic reconstruction.

Based on the chloroplast phylogenomic relationships described above, we found that the *Agapetes* clade was nested within *Vaccinium* (**Figures 2a**, **S4a**). This appears to support merging both genera into a single taxonomic unit. To further validate this finding, we expanded our DNA sampling to include nuclear genome-derived DNA of eight *Agapetes* and seven *Vaccinium* species (**Supplementary Table S2**), targeting the ITS regions (ITS1, 5.8S rDNA, and ITS2)—widely used markers for nuclear phylogenetics in plants (Liu et al., 2006).

Following multiple sequence alignment (**Supplementary Figure S3**), we reconstructed nuclear DNA phylogenies using both ML and BI methods for full ITS, ITS1, and ITS2 (**Figures 2b**, **S4b, S5**). Despite low statistical support, the phylogenetic results showed topological stability across all datasets and analytical methods: *Agapetes* and *Vaccinium* species are paraphyletic within a shared monophyletic clade (**Figures 2**, **S4, S5**). Both the cp genome and ITS phylogenies exhibit small cumulative branch lengths between the species of *Agapetes* and *Vaccinium*, indicating that there are not large differences among these species. Although chloroplast genomes and ITS-based nuclear sequences reconstructed nested monophyletic clade for species of *Agapetes* and *Vaccinium*, these findings require further validation with large-scale sampling across the genus. Hierarchical clustering based on RSCU, abundance of SSRs, and dispersed repeats also supported the close relationship between *Agapetes* and *Vaccinium* species (**Figures 5b, d**; **S6** **;** **Supplementary Tables S8, S13, S14**).

The three *Agapetes* species (*A. malipoensis*, *A. guangxiensis*, and *A. obovata*) formed a monophyletic clade in the chloroplast phylogenomics, but they were dispersed across three distinct clades in the ITS-based nuclear phylogeny (**Figures 2**, **S4, S5**). The discordance between ITS and cpDNA phylogenies likely stems from distinct evolutionary histories of chloroplasts (maternally inherited organellar genomes) versus nuclear DNA (biparentally inherited). Furthermore, in rapidly radiating lineages, species-level phylogenies often cannot be accurately reconstructed from single-gene analyses due to processes like hybridization or incomplete lineage sorting (ILS) (Huerta-Sánchez et al., 2014; Edelman et al., 2019; Shen et al., 2021). The overlap in flowering periods of species from the two genera may facilitate hybridization between them (**Supplementary Table S9**). Another potential source of this discordance is interplastomic recombination (Li et al., 2025). Of course, the low statistical support of ITS, ITS1, and ITS2 phylogenies could also be caused by hybridization, ILS, or limited sequence variations.

Further comparative analyses to chloroplast genomes revealed minimal disparities in genome length, GC content, and encoded genes among species of *Agapetes* and *Vaccinium* (**Figures 3**, **4** **;** **Supplementary Tables S11, S12**). SSRs are extensively distributed across chloroplast genomes (Xia et al., 2022). Our study found that A/T-type mononucleotide repeats were prevalent among the 15 investigated plant species (**Figures 5a, b**; **Supplementary Table S13**), consistent with prior research indicating their predominance and rarity of C/G repeats (Kuang et al., 2011). The number of SSRs in the chloroplast genomes of the three *Agapetes* is similar, with a negligible difference from the quantity found in *Vaccinium* (**Figures 5a, b**; **Supplementary Table S13**). Moreover, both genera contain a higher number of hexanucleotide repeat types. In contrast, there is a large difference in the total number of SSRs between species of *Agapetes*–*Vaccinium* and other species, with other species either lacking or containing only one type of hexanucleotide repeat. This result indicates that the SSR composition in *Agapetes* is similar to that in *Vaccinium*.

Previous research studies have shown that long repeats are prevalent in genomes, significantly influencing gene expression, regulation, and plant systematics research (Cavalier-Smith, 2002). We identified a significant number of forward and palindromic repetitive sequences in the chloroplast genomes of three *Agapetes* and *Vaccinium*, with a total count exceeding 1,000, which is much higher than in other genera and species (**Figures 5c, d**; **Supplementary Table S14**). Furthermore, both *Agapetes* and *Vaccinium* exhibited tandem repeats with similar locations and repeat unit size. The multiple sequence alignment of the 219-bp repeat consensus sequence from *Agapetes* and *Vaccinium* supports the close evolutionary relationship between the two genera (**Figure 5e**).

The contraction and expansion of IR boundaries are ubiquitous in the evolutionary history of plants, often leading to differences in chloroplast genome size among different species (Kim and Lee, 2004). The three *Agapetes* and *Vaccinium* showed minor differences in the variation characteristics within IR boundaries (**Figure 6**). This indicates that there are no substantial differences between the two genera in this character at this sampling level.

Despite the overall conservation in chloroplast genome evolution (**Figure 3**), IR boundary regions and repetitive sequences (SSRs, tandem repeats, dispersed repeats) exhibit accelerated evolutionary rate (Ané et al., 2005). The intermixed pattern between *Agapetes* and *Vaccinium* species was not only revealed in chloroplast genomic and ITS phylogenetic analyses, but was also supported by hierarchical clustering based on RSCU and the abundance of rapidly evolving features such as SSRs and dispersed repeats. Species of the two genera exhibited no large differences in other key chloroplast genomic features, including the structure of coding and non-coding regions, as well as the dynamically evolving IR boundaries. Given the persistent morphological controversies between *Agapetes* and *Vaccinium*, coupled with our molecular evidence, we propose to merge the two genera into a single taxonomic unit. *Vaccinium* comprises significantly more species (ca. 500) than *Agapetes* (ca. 115) (POWO, 2025). Most importantly, *Vaccinium* was published earlier (Linnaeus, 1753) than *Agapetes* (D. Don ex G. Don, 1834) (POWO, 2025). In accordance with the principle of priority (ICN Art. 11), we therefore propose to treat *Agapetes* as a synonym of *Vaccinium*. However, before any formal taxonomic change can be enacted, large-scale sampling of cp genomes from numerous species (especially undersampled Asian tropical species) should be conducted, where the cp genomes provided in this study will be valuable references.

This study has limitations requiring further improvement. Given the extensive harvesting of *Agapetes* species for medicinal purposes by local communities, we could only obtain three readily accessible species, each represented by a single individual. Future efforts will implement optimized sampling strategies to collect multiple individuals across taxa, while advancing nuclear genome-scale investigations. By employing hundreds of nuclear genes for coalescent-based species tree reconstruction, we aim to minimize confounding effects from hybridization or incomplete lineage sorting. Concurrently, we will explore the genomic foundations of medicinal traits of *Agapetes* through comparative transcriptomics and functionally validate key biosynthetic genes via qPCR profiling across tissues, or transgenic approaches using CDB transformation systems (Cao et al., 2023).

## 5 Conclusion

To resolve the persistent controversy regarding the taxonomic circumscription of *Agapetes* and *Vaccinium*, we completed the sequencing, assembly, phylogenetic, structural, and characteristic analyses of the chloroplast genomes of three *Agapetes* species for the first time. According to phylogenetic results, the two genera are not reciprocally monophyletic. In the chloroplast phylogenomic analysis, the three species of *Agapetes* formed a monophyletic clade nested within *Vaccinium*. The ITS phylogeny further revealed a paraphyletic phylogenetic pattern among species of *Agapetes* and *Vaccinium*. Moreover, the two genera also could not be distinguished by various chloroplast genomic characteristics, such as GC content, coding/non-coding region proportion, RSCU, IR boundary shifts, and rapidly evolving repetitive sequences. Based on these findings, we propose to merge the two genera into a single taxonomic unit after large-scale sampling of the cp genome in *Vaccinieae*.

## Data Availability

The datasets presented in this study can be found in online repositories. The names of the repository/repositories and accession number(s) can be found in the article/**Supplementary Material**.
