# Supplementary material for: Chloroplast phylogenomics provides new evidence for reevaluating the taxonomic placement of medicinal Agapetes
Source: Front Plant Sci. 2025 Oct 22;16:1586413. doi: 10.3389/fpls.2025.1586413 (PMC12586127; doi:10.3389/fpls.2025.1586413)
Supplement: Supplementary file 1 [file DataSheet1.docx]

Supplementary Material

1. Supplementary Figures and Tables
   1. Supplementary Figures
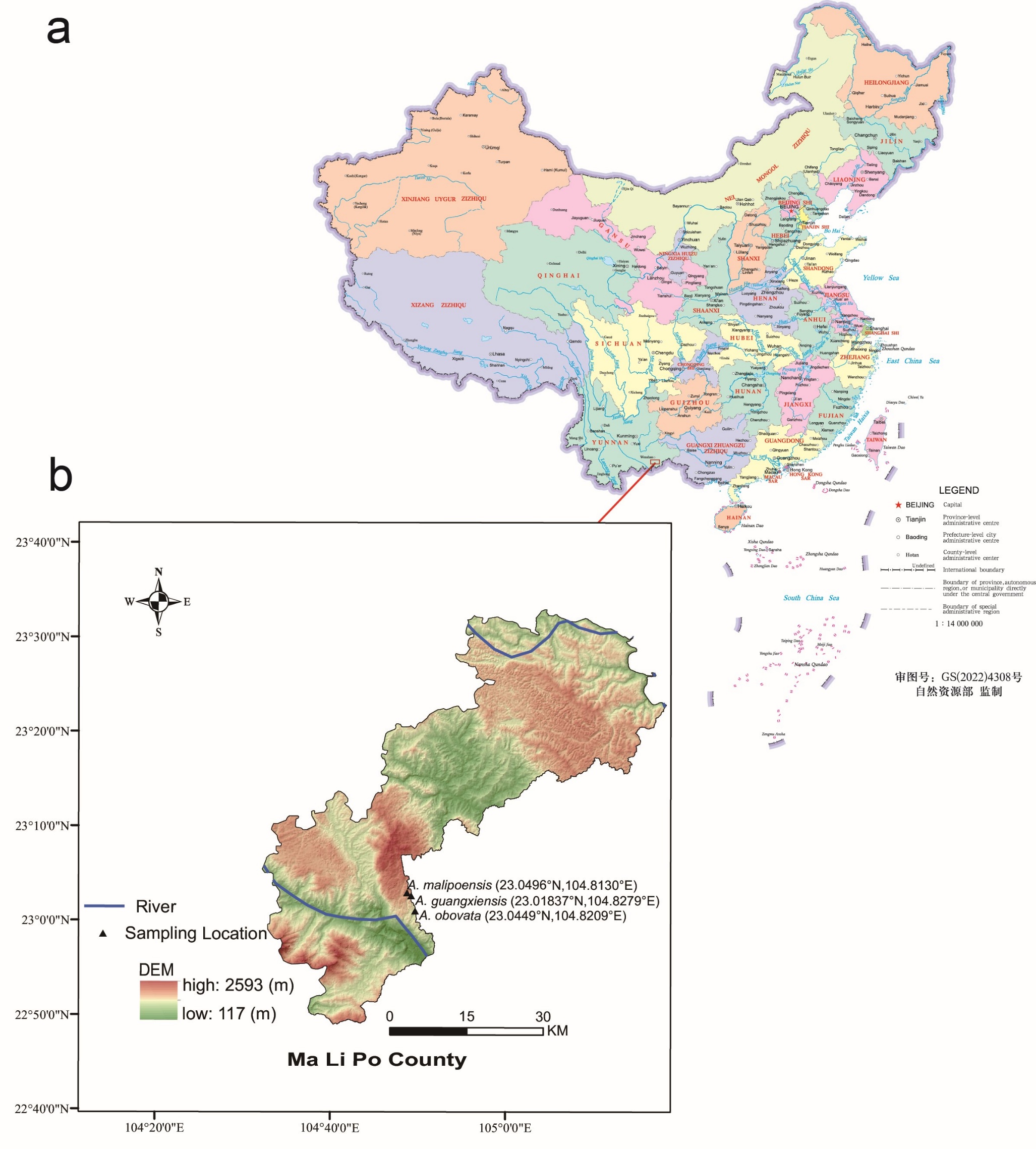


**Figure S1. Sample collection sites.** a). Map of China, from http://211.159.153.75/, review number: GS(2022)4308. b). Map of Ma Li Po County.


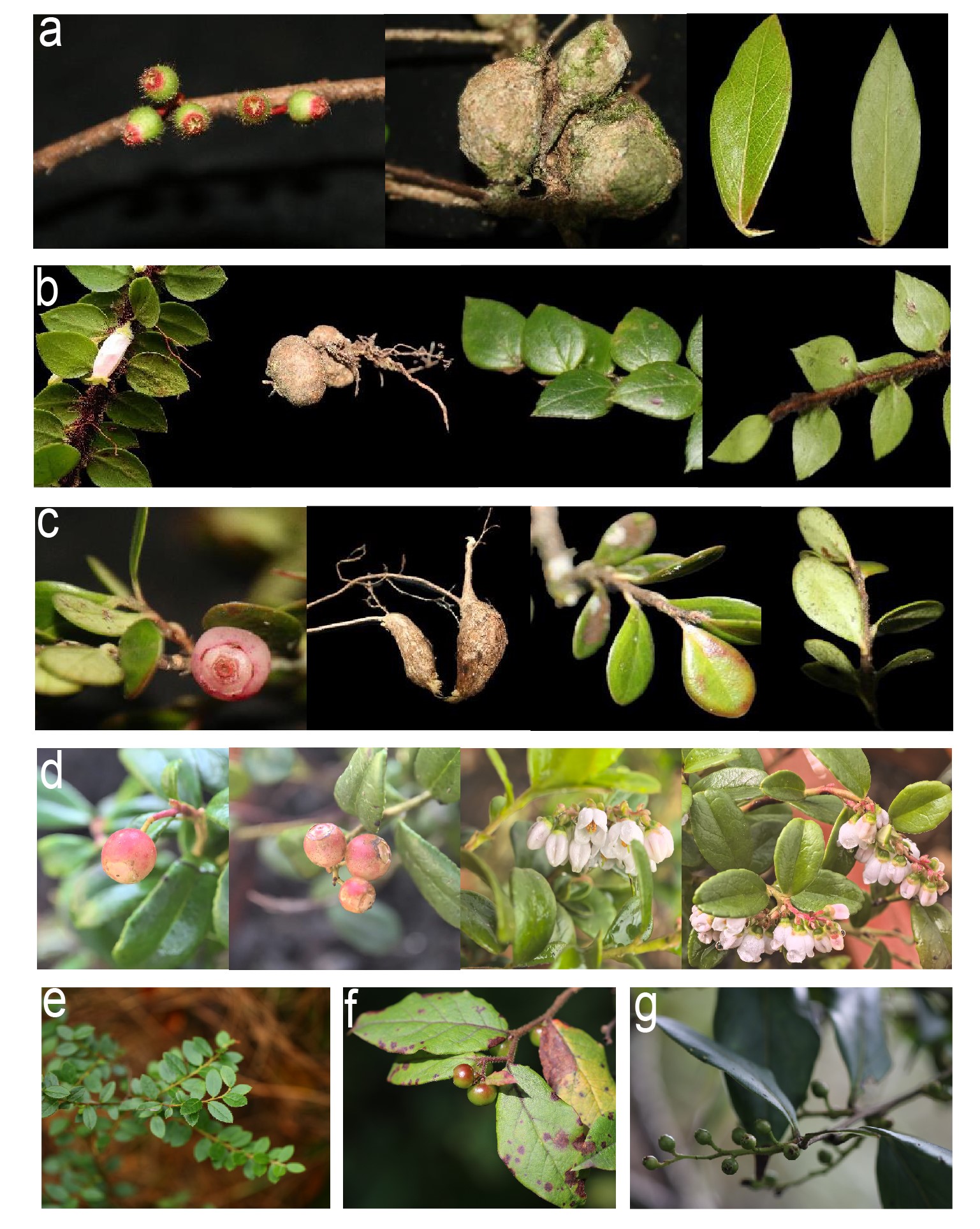


**Figure S2. The morphological characteristic of *Agapetes* and** ***Vaccinium*.** a). *A. malipoensis.* b). *A. guangxiensis*. c). *A. obovata*. d). *V. vitis-idaea*. e). *V. fragile*. f). *V. henryi*. g). *V. dunnianum*. Photos of *Agapetes* species were taken by the authors, *V. vitis-idaea* by Dr. Su Zhang from School of Forestry, Beijing Forestry University, the other *Vaccinium* species by Dr. Chao Zhang from School of Life Sciences, Guizhou Normal University.


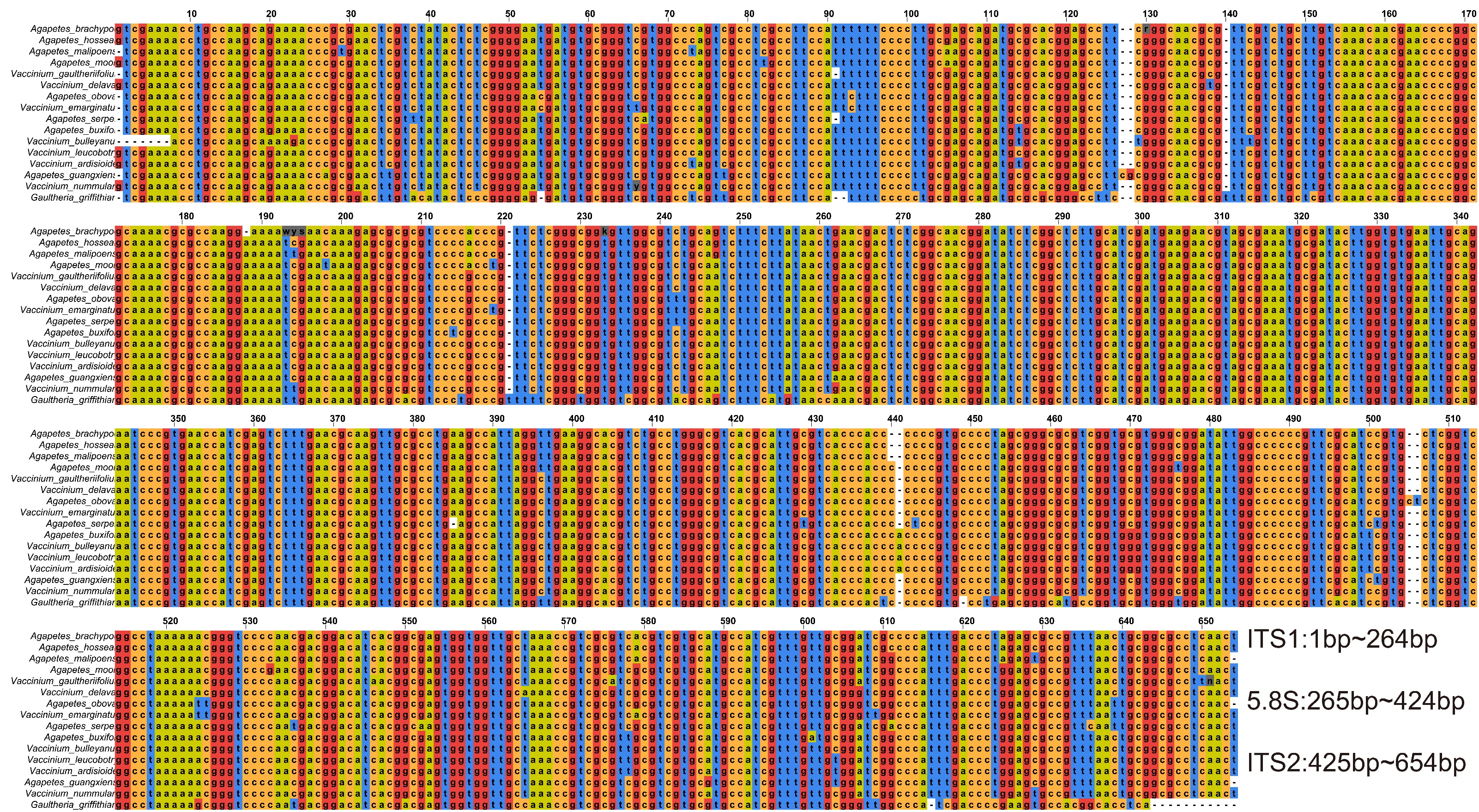


**Figure S3. Multiple sequence alignment of ITS regions.** ITS1 (bp 1-264), 5.8S rRNA (bp 265-424), and ITS2 (bp 425-654).


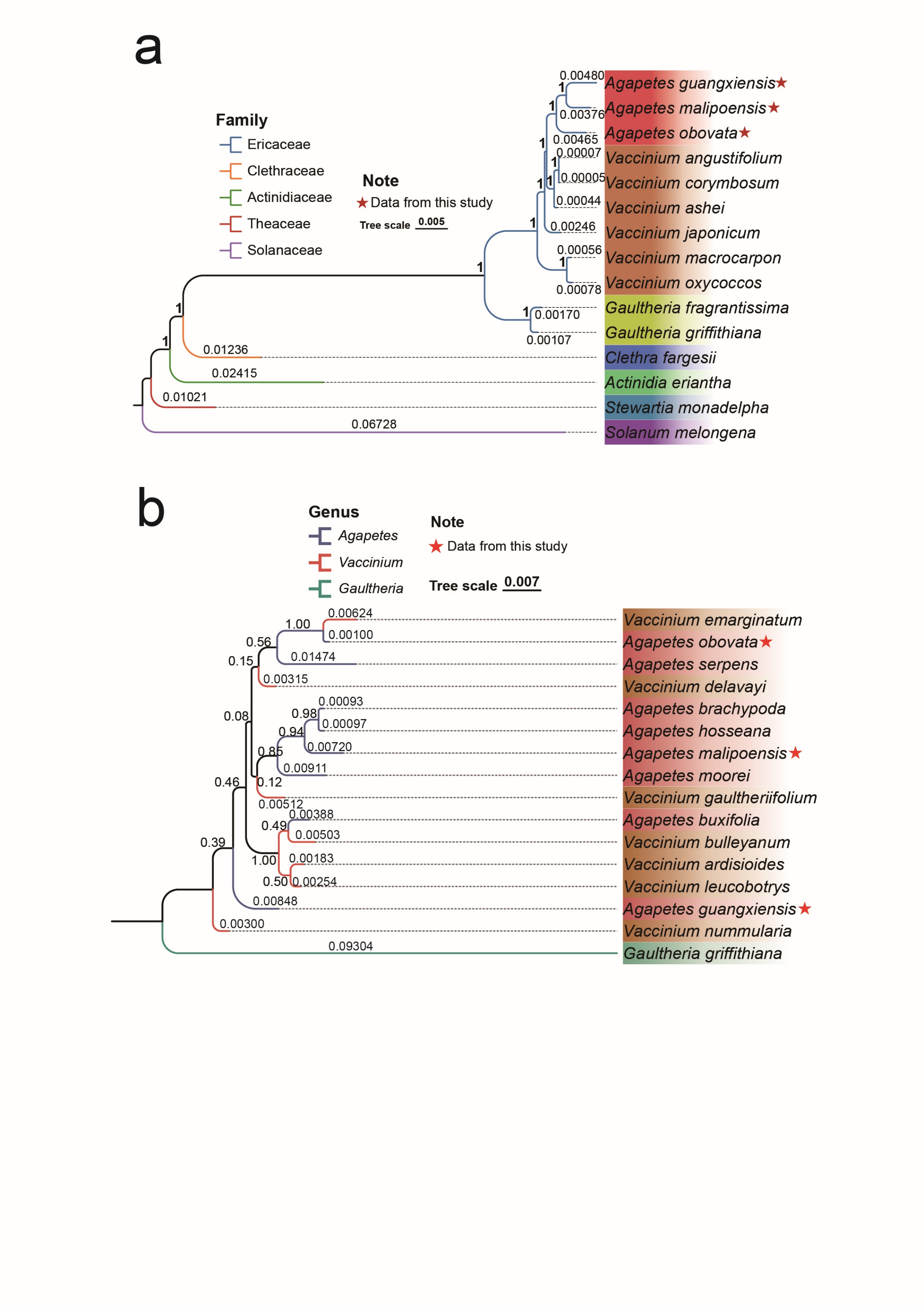


**Figure S4. Phylogenetic relationships of *Agapetes* and closely related species.** a). Protein-coding genes CDs sequences tree constructed by Bayesian inference (BI) methods with the posterior probabilities of BI. b). ITS sequences tree constructed by Bayesian inference (BI) methods with the posterior probabilities of BI.


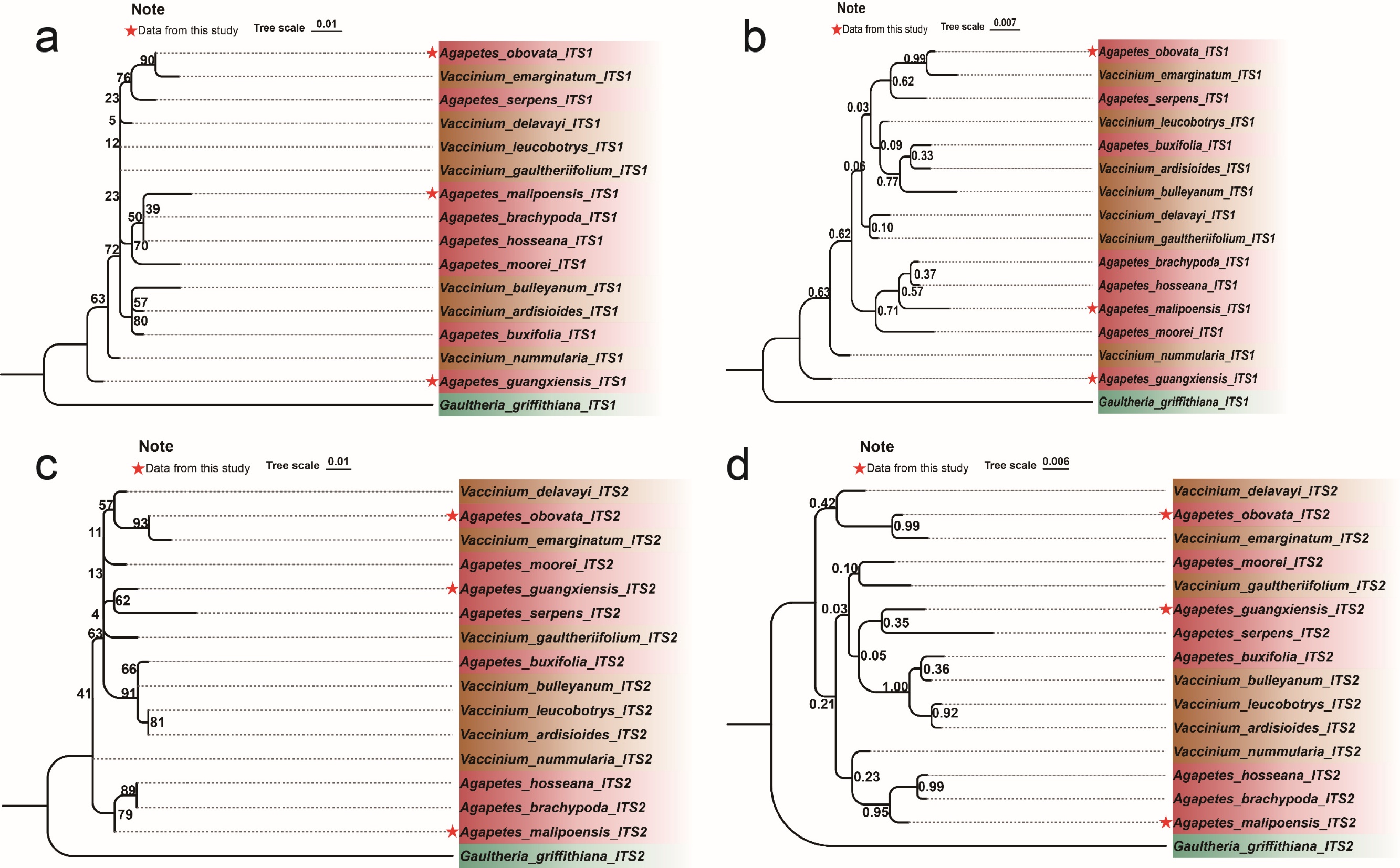


**Figure S5. Phylogenetic relationships of *Agapetes* and closely related species.** a). Phylogenetic tree of ITS1 sequences constructed by maximum likelihood (ML) method with bootstrap support values (BS). b). ITS1 sequences tree constructed by Bayesian inference (BI) methods with the posterior probabilities of BI. c). Phylogenetic tree of ITS2 sequences constructed by maximum likelihood (ML) method with bootstrap support values (BS). d). ITS2 sequences tree constructed by Bayesian inference (BI) methods with the posterior probabilities of BI.


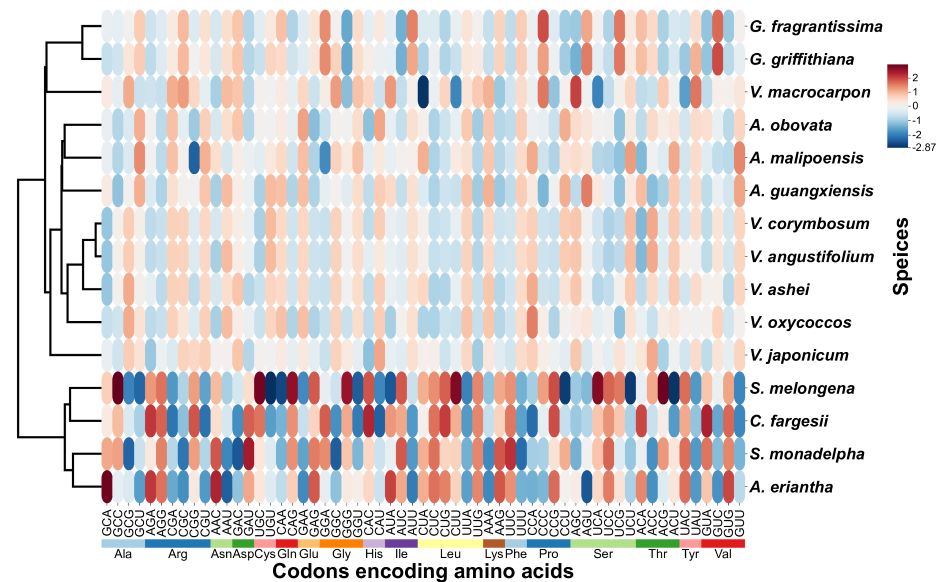


**Figure S6.** **Thirteen species of Codon Usage Bias**. RSCU values for codons.


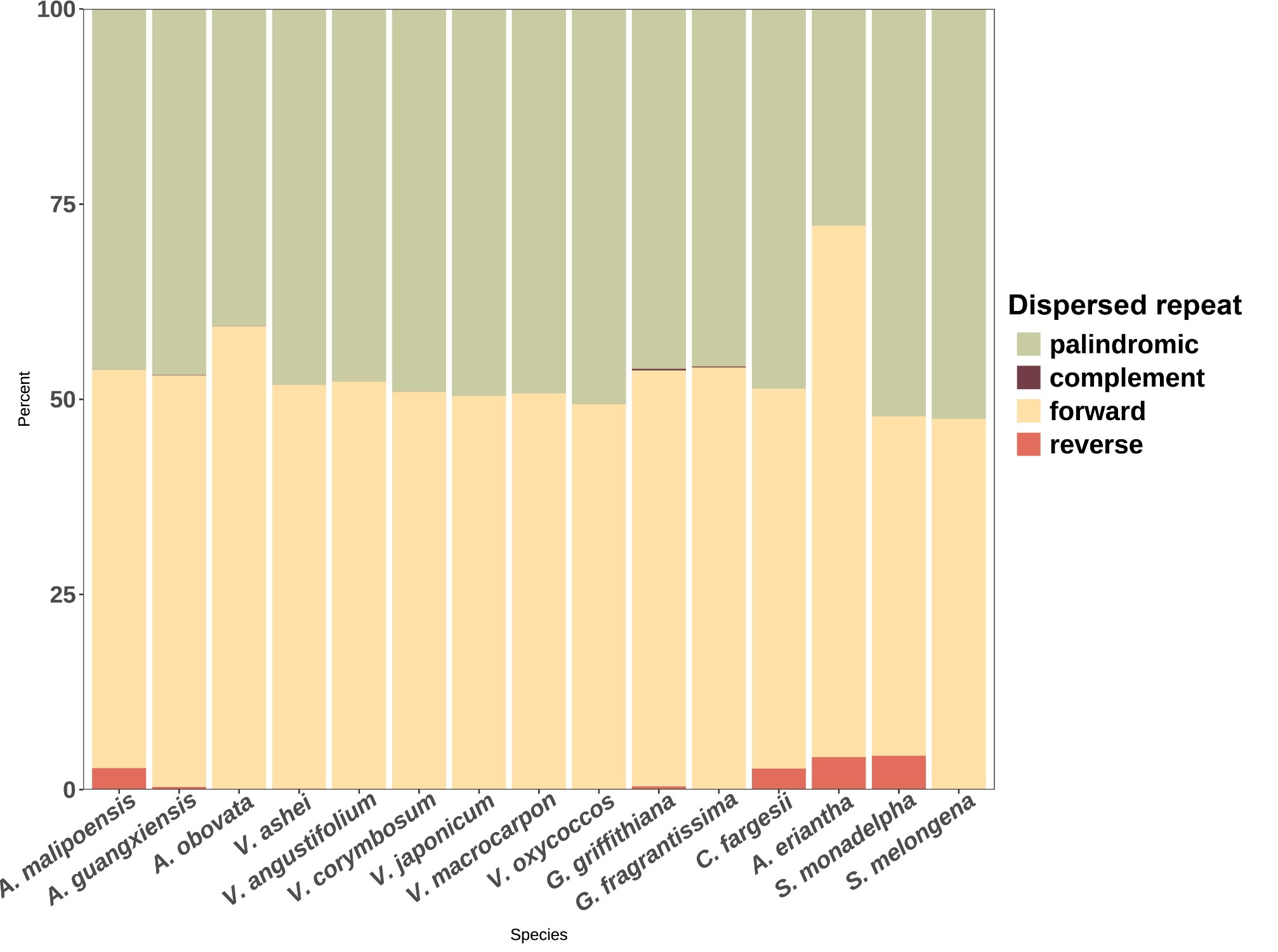


**Figure S7. Proportion of different type of dispersed repeats.**


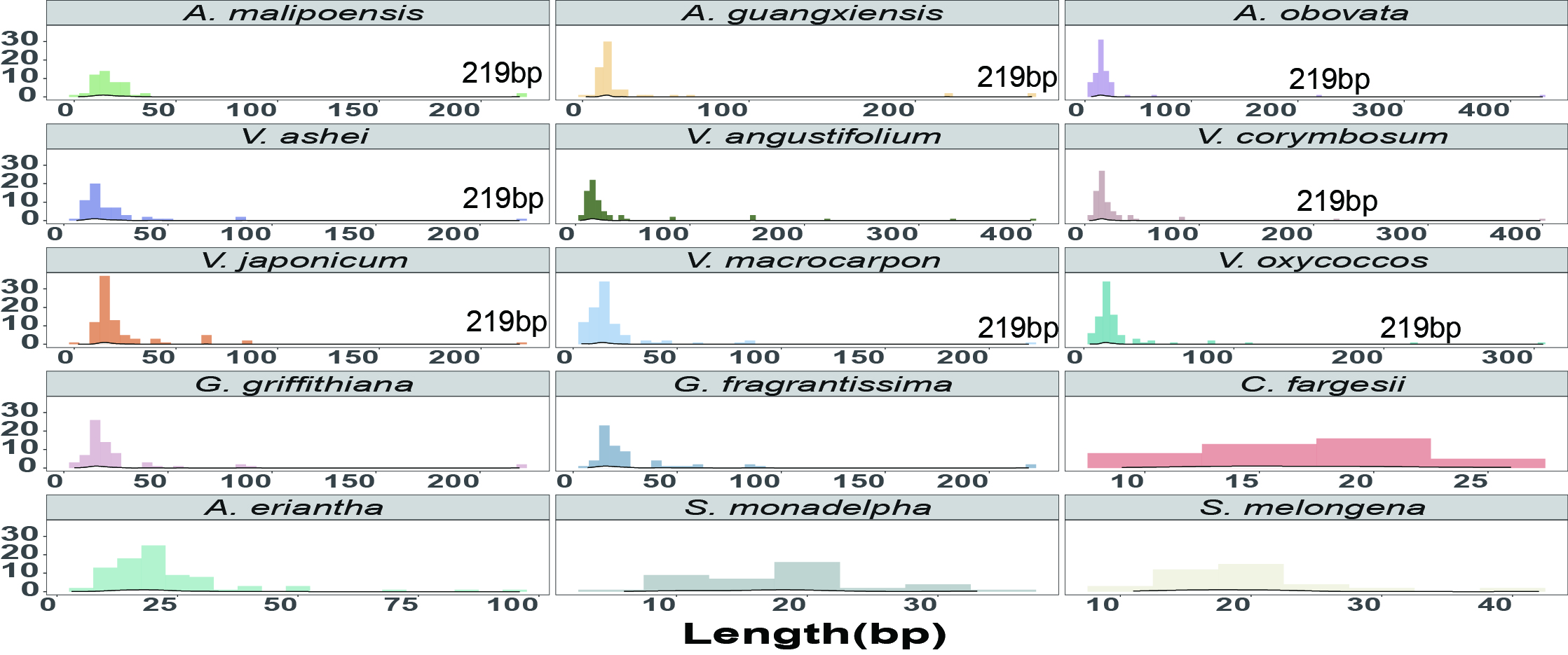


**Figure S8. Numbers of Tandem sequences by length.**

- 1. Supplementary Tables

**Table S1. Complete Chloroplast genome data resources of the genomes used in this study**

| **Family** | **Species** | **Source** | | **Accession number** | | **Reference** | | **Abridge** | |
| --- | --- | --- | --- | --- | --- | --- | --- | --- | --- |
| Ericaceae | *Agapetes malipoensis* | NCBI | MZ522113.1 | | This study | | *A. malipoensis* | |  |
| Ericaceae | *Agapetes guangxiensis* | NCBI | PP765882.1 | | This study | | *A. guangxiensis* | |  |
| Ericaceae | *Agapetes obovata* | NCBI | PP765883.1 | | This study | | *A. obovata* | |  |
| Ericaceae | *Vaccinium ashei* | NCBI | KM269111.1 | | (Kim et al., 2020) | | *V. ashei* | |  |
| Ericaceae | *Vaccinium angustifolium* | NCBI | JQ248601.1 | | (Fahrenkrog et al., 2022) | | *V. macrocarpon* | |  |
| Ericaceae | *Vaccinium corymbosum* | NCBI | OM791344 | | (Fahrenkrog et al., 2022) | | *V. angustifolium* | |  |
| Ericaceae | *Vaccinium japonicum* | NCBI | OQ311354.1 | | (López et al., 2023) | | *V. japonicum* | |  |
| Ericaceae | *Vaccinium macrocarpon* | NCBI | BK061167.1 | | (Fahrenkrog et al., 2022) | | *V. corymbosum* | |  |
| Ericaceae | *Vaccinium oxycoccos* | NCBI | NC_081496.1 | | (Qiao et al., 2023) | | *V. oxycoccos* | |  |
| Ericaceae | *Gaultheria griffithiana* | NCBI | OM048894.1 | | (Li et al., 2021) | | *G. griffithiana* | |  |
| Ericaceae | *Gaultheria fragrantissima* | NCBI | MW563322.1 | | (Yan-Ling et al., 2021) | | *G. fragrantissima* | |  |
| Clethraceae | *Clethra fargesii* | NCBI | MT742578.1 | | (Ding et al., 2021) | | *C. fargesii* | |  |
| Actinidiaceae | *Actinidia eriantha* | NCBI | MZ959065.1 | | (Tang et al., 2019) | | *A. eriantha* | |  |
| Theaceae | *Stewartia monadelpha* | NCBI | NC_041468.1 | | (Lin et al., 2019) | | *S. monadelpha* | |  |
| Solanaceae | *Solanum melongena* | NCBI | MF818319.1 | | (Knapp et al., 2013) | | *S. melongena* | |  |

**Table S2. Internal Transcribed Spacer data resources used in this study**

| **Family** | **Species** | **Source** | **Accession number** | **Reference** |
| --- | --- | --- | --- | --- |
| Ericaceae | *Agapetes obovata* | NCBI | PQ303664 | This study |
| Ericaceae | *Agapetes malipoensis* | NCBI | PQ303665 | This study |
| Ericaceae | *Agapetes guangxiensis* | NCBI | PQ303666 | This study |
| Ericaceae | *Agapetes buxifolia* | NCBI | AF382650 | (Kron et al., 2002) |
| Ericaceae | *Agapetes serpens* | NCBI | AF382653 | (Kron et al., 2002) |
| Ericaceae | *Agapetes brachypoda* | NCBI | KM209367 | (Matuszak, 2015) |
| Ericaceae | *Agapetes hosseana* | NCBI | KM209369 | (Matuszak, 2015) |
| Ericaceae | *Agapetes moorei* | NCBI | KM209373 | (Matuszak, 2015) |
| Ericaceae | *Vaccinium ardisioides* | NCBI | KM209421 | (Matuszak, 2015) |
| Ericaceae | *Vaccinium bulleyanum* | NCBI | KM209429 | (Matuszak, 2015) |
| Ericaceae | *Vaccinium delavayi* | NCBI | KM209438 | (Matuszak, 2015) |
| Ericaceae | *Vaccinium leucobotrys* | NCBI | KM209451 | (Matuszak, 2015) |
| Ericaceae | *Vaccinium nummularia* | NCBI | KM20945 | (Matuszak, 2015) |
| Ericaceae | *Vaccinium emarginatum* | NCBI | AB623181 | (Matuszak, 2015) |
| Ericaceae | *Vaccinium gaultheriifolium* | NCBI | AF382726 | (Kron et al., 2002) |
| Ericaceae | *Gaultheria griffithiana* | NCBI | JF976365 | (Li et al., 2011) |

**Table S3. The chloroplast genome sequencing results of *A. malipoensis*, *A. guangxiensis*, and *A. obovata*.**

| **species** | **BaseSum(bp)** | **depth(x)** |
| --- | --- | --- |
| *A.malipoensis* | 5670142200 | 2275.45 |
| *A.guangxiensis* | 12487132800 | 1391.91 |
| *A.obovata* | 10368796800 | 3547.42 |

**Table S4. Statistics of the chloroplast genomes of *Agapetes* and other 12 species**

| **Species** | **Length (bp)** | **GC content (%)** | **LSC (bp)** | **IR (bp)** | **SSC (bp)** |
| --- | --- | --- | --- | --- | --- |
| *A. malipoensis* | 172729 | 36.67% | 105281 | 32209 | 3030 |
| *A. guangxiensis* | 176291 | 36.47% | 105187 | 34046 | 3012 |
| *A. obovata* | 180574 | 36.65% | 106560 | 35473 | 3068 |
| *V. ashei* | 176115 | 36.81% | 106029 | 33541 | 3004 |
| *V. angustifolium* | 176095 | 36.80% | 104591 | 34238 | 3028 |
| *V. corymbosum* | 182334 | 36.80% | 107607 | 35865 | 2997 |
| *V. japonicum* | 187213 | 36.74% | 104637 | 39788 | 3000 |
| *V. macrocarpon* | 186057 | 36.80% | 105714 | 38658 | 3027 |
| *V. oxycoccos* | 177088 | 36.74% | 104139 | 34959 | 3031 |
| *G. griffithiana* | 178045 | 36.63% | 107415 | 33494 | 3642 |
| *G. fragrantissima* | 176196 | 36.60% | 107747 | 32470 | 3059 |
| *C. fargesii* | 157486 | 37.34% | 87034 | 25980 | 18492 |
| *A. eriantha* | 156964 | 37.19% | 90231 | 23096 | 20541 |
| *S. monadelpha* | 158447 | 37.25% | 87769 | 26272 | 18134 |
| *S. melongena* | 155569 | 37.71% | 86182 | 25443 | 18501 |

**Table S5. Groups of genes within the *A. malipoensis* chloroplast genome**

| **Category** | **Gene group** | **Gene name** |
| --- | --- | --- |
| Photosynthesis | Subunits of photosystem I | psaA,psaB,psaC(2),psaI,psaJ |
|  | Subunits of photosystem II | psbA,psbB,psbC,psbD,psbE,psbF,psbH,psbI,psbJ,psbK,psbL,psbM,psbN,psbT,psbZ |
|  | Subunits of NADH dehydrogenase | ndhA*(2),ndhB*,ndhC,ndhD(2),ndhE(2),ndhF,ndhG(2),ndhH(2),ndhI(2),ndhJ,ndhK |
|  | Subunits of cytochrome b/f complex | petA,petB*,petD*,petG,petL,petN |
|  | Subunits of ATP synthase | atpA,atpB,atpE,atpF*,atpH,atpI |
|  | Large subunit of rubisco | rbcL |
| Self-replication | Proteins of large ribosomal subunit | rpl14,rpl16*,rpl2,rpl20,rpl22,rpl23,rpl32(2),rpl33,rpl36 |
|  | Proteins of small ribosomal subunit | rps11,rps12**,rps14,rps15(2),rps16*(2),rps18,rps19,rps2,rps3,rps4,rps7,rps8 |
|  | Subunits of RNA polymerase | rpoA,rpoB,rpoC1*,rpoC2 |
|  | Ribosomal RNAs | rrn16(2),rrn23(2),rrn4.5(2),rrn5(2) |
|  | Transfer RNAs | trnA-UGC*(2),trnC-GCA,trnD-GUC,trnE-UUC,trnF-GAA,trnG-GCC(2),trnG-UCC*,trnH-GUG(2),trnI-CAU,trnI-GAU*(2),trnK-UUU*,trnL-CAA,trnL-UAA*,trnL-UAG(2),trnM-CAU,trnN-GUU(2),trnP-UGG,trnQ-UUG,trnR-ACG(2),trnR-UCU,trnS-GGA(2),trnS-UGA,trnT-GGU,trnT-UGU,trnV-GAC,trnV-UAC*,trnW-CCA,trnY-GUA,trnfM-CAU(3) |
| Other genes | Maturase | matK |
|  | Envelope membrane protein | cemA |
|  |  |  |
|  | c-type cytochrome synthesis gene | ccsA(2) |
| Genes of unknown function | Conserved hypothetical chloroplast ORF | ycf3**,ycf4 |
|  | | |

NOTE:Gene*:Gene with one intron, Gene**:Gene with two introns,#Gene:Pseudo gene,Gene(2):Number of copies of multi-copy genes.

**Table S6. Groups of genes within the *A. guangxiensis* chloroplast genome**

| **Category** | **Gene group** | **Gene name** |
| --- | --- | --- |
| Photosynthesis | Subunits of photosystem I | psaA,psaB,psaC(2),psaI,psaJ |
|  | Subunits of photosystem II | psbA,psbB,psbC,psbD,psbE,psbF,psbH,psbI,psbJ,psbK,psbL,psbM,psbN,psbT,psbZ |
|  | Subunits of NADH dehydrogenase | ndhA*(2),ndhB*,ndhC,ndhD(2),ndhE(2),ndhF,ndhG(2),ndhH(2),ndhI(2),ndhJ,ndhK |
|  | Subunits of cytochrome b/f complex | petA,petB*,petD*,petG,petL,petN |
|  | Subunits of ATP synthase | atpA,atpB,atpE,atpF*,atpH,atpI |
|  | Large subunit of rubisco | rbcL |
| Self-replication | Proteins of large ribosomal subunit | rpl14,rpl16*,rpl2,rpl20,rpl22,rpl23,rpl32(2),rpl33,rpl36 |
|  | Proteins of small ribosomal subunit | rps11,rps12**,rps14,rps15(2),rps16*(2),rps18,rps19,rps2,rps3,rps4,rps7,rps8 |
|  | Subunits of RNA polymerase | rpoA,rpoB,rpoC1*,rpoC2 |
|  | Ribosomal RNAs | rrn16(2),rrn23(2),rrn4.5(2),rrn5(2) |
|  | Transfer RNAs | trnA-UGC*(2),trnC-GCA,trnD-GUC,trnE-UUC,trnF-GAA,trnG-GCC,trnG-UCC*,trnH-GUG(2),trnI-CAU,trnI-GAU*(2),trnK-UUU*,trnL-CAA,trnL-UAA*,trnL-UAG(2),trnM-CAU,trnN-GUU(2),trnP-UGG,trnQ-UUG,trnR-ACG(2),trnR-UCU,trnS-GCU,trnS-GGA,trnS-UGA,trnT-GGU,trnT-UGU,trnV-GAC,trnV-UAC*,trnW-CCA,trnY-GUA,trnfM-CAU(2) |
| Other genes | Maturase | matK |
|  | Envelope membrane protein | cemA |
|  |  |  |
|  | c-type cytochrome synthesis gene | ccsA(2) |
| Genes of unknown function | Conserved hypothetical chloroplast ORF | ycf3**,ycf4 |
|  | | |

NOTE:Gene*:Gene with one intron, Gene**:Gene with two introns,#Gene:Pseudo gene,Gene(2):Number of copies of multi-copy genes.

**Table S7. Groups of genes within the *A. obovata* chloroplast genome**

| **Category** | **Gene group** | **Gene name** |
| --- | --- | --- |
| Photosynthesis | Subunits of photosystem I | psaA,psaB,psaC(2),psaI,psaJ |
|  | Subunits of photosystem II | psbA,psbB,psbC,psbD,psbE,psbF,psbH,psbI,psbJ,psbK,psbL,psbM,psbN,psbT,psbZ |
|  | Subunits of NADH dehydrogenase | ndhA*(2),ndhB*,ndhC,ndhD(2),ndhE(2),ndhF,ndhG(2),ndhH(2),ndhI(2),ndhJ,ndhK |
|  | Subunits of cytochrome b/f complex | petA,petB*,petD*,petG,petL,petN |
|  | Subunits of ATP synthase | atpA,atpB,atpE,atpF*,atpH,atpI |
|  | Large subunit of rubisco | rbcL |
| Self-replication | Proteins of large ribosomal subunit | rpl14,rpl16*,rpl2,rpl20,rpl22,rpl23,rpl32(2),rpl33,rpl36 |
|  | Proteins of small ribosomal subunit | rps11,rps12**,rps14,rps15(2),rps16*(2),rps18,rps19,rps2,rps3,rps4,rps7,rps8 |
|  | Subunits of RNA polymerase | rpoA,rpoB,rpoC1*,rpoC2 |
|  | Ribosomal RNAs | rrn16(2),rrn23(2),rrn4.5(2),rrn5(2) |
|  | Transfer RNAs | trnA-UGC*(2),trnC-GCA,trnD-GUC,trnE-UUC,trnF-GAA,trnG-GCC,trnG-UCC*,trnH-GUG(2),trnI-CAU,trnI-GAU*(2),trnK-UUU*,trnL-CAA,trnL-UAA*,trnL-UAG(2),trnM-CAU,trnN-GUU(2),trnP-UGG,trnQ-UUG,trnR-ACG(2),trnR-UCU,trnS-GCU,trnS-GGA,trnS-UGA,trnT-GGU,trnT-UGU,trnV-GAC,trnV-UAC*,trnW-CCA,trnY-GUA,trnfM-CAU(2) |
| Other genes | Maturase | matK |
|  | Envelope membrane protein | cemA |
|  |  |  |
|  | c-type cytochrome synthesis gene | ccsA(2) |
| Genes of unknown function | Conserved hypothetical chloroplast ORF | ycf3**,ycf4 |
|  | | |

NOTE:Gene*:Gene with one intron, Gene**:Gene with two introns,#Gene:Pseudo gene,Gene(2):Number of copies of multi-copy genes.

**Table S8. Statistics on the Relative Synonymous Codon Usage (RSCU) for the chloroplast genomes of *Agapetes* and 12 other species.**

| **Amino Acids** | **Codon** | ***A. malipoensis*** | ***A. guangxiensis*** | ***A. obovata*** | ***V. ashei*** | ***V. angustifolium*** | ***V. corymbosum*** | ***V. japonicum*** | ***V. macrocarpon*** | ***V. oxycoccos*** | ***G. griffithiana*** | ***G. fragrantissima*** | ***C. fargesii*** | ***A. eriantha*** | ***S. monadelpha*** | ***S. melongena*** |
| --- | --- | --- | --- | --- | --- | --- | --- | --- | --- | --- | --- | --- | --- | --- | --- | --- |
| Ala | GCA | 1.12 | 1.13 | 1.12 | 1.1 | 1.1 | 1.1 | 1.12 | 1.13 | 1.11 | 1.11 | 1.12 | 1.13 | 1.19 | 1.15 | 1.14 |
| Ala | GCC | 0.56 | 0.55 | 0.56 | 0.6 | 0.59 | 0.59 | 0.57 | 0.58 | 0.57 | 0.57 | 0.58 | 0.62 | 0.58 | 0.63 | 0.69 |
| Ala | GCG | 0.43 | 0.44 | 0.43 | 0.5 | 0.46 | 0.46 | 0.46 | 0.47 | 0.47 | 0.45 | 0.44 | 0.43 | 0.43 | 0.38 | 0.39 |
| Ala | GCU | 1.89 | 1.88 | 1.88 | 1.9 | 1.85 | 1.85 | 1.86 | 1.83 | 1.86 | 1.87 | 1.87 | 1.82 | 1.8 | 1.83 | 1.78 |
| Arg | AGA | 1.71 | 1.69 | 1.71 | 1.7 | 1.67 | 1.67 | 1.65 | 1.68 | 1.67 | 1.67 | 1.68 | 1.84 | 1.84 | 1.73 | 1.79 |
| Arg | AGG | 0.45 | 0.44 | 0.45 | 0.5 | 0.46 | 0.46 | 0.5 | 0.46 | 0.46 | 0.48 | 0.48 | 0.64 | 0.63 | 0.62 | 0.63 |
| Arg | CGA | 1.55 | 1.53 | 1.55 | 1.5 | 1.54 | 1.54 | 1.49 | 1.56 | 1.54 | 1.52 | 1.52 | 1.41 | 1.44 | 1.47 | 1.43 |
| Arg | CGC | 0.4 | 0.4 | 0.39 | 0.4 | 0.42 | 0.42 | 0.42 | 0.44 | 0.41 | 0.43 | 0.43 | 0.36 | 0.34 | 0.33 | 0.37 |
| Arg | CGG | 0.33 | 0.4 | 0.37 | 0.4 | 0.39 | 0.39 | 0.42 | 0.42 | 0.39 | 0.4 | 0.39 | 0.46 | 0.41 | 0.44 | 0.44 |
| Arg | CGU | 1.56 | 1.54 | 1.53 | 1.5 | 1.52 | 1.52 | 1.53 | 1.45 | 1.53 | 1.5 | 1.51 | 1.29 | 1.33 | 1.4 | 1.34 |
| Asn | AAC | 0.45 | 0.42 | 0.43 | 0.4 | 0.42 | 0.43 | 0.44 | 0.42 | 0.42 | 0.43 | 0.43 | 0.45 | 0.49 | 0.48 | 0.46 |
| Asn | AAU | 1.55 | 1.58 | 1.57 | 1.6 | 1.58 | 1.57 | 1.56 | 1.58 | 1.58 | 1.57 | 1.57 | 1.55 | 1.51 | 1.52 | 1.54 |
| Asp | GAC | 0.42 | 0.42 | 0.43 | 0.4 | 0.42 | 0.42 | 0.43 | 0.43 | 0.42 | 0.43 | 0.43 | 0.39 | 0.4 | 0.38 | 0.41 |
| Asp | GAU | 1.58 | 1.58 | 1.57 | 1.6 | 1.58 | 1.58 | 1.57 | 1.57 | 1.58 | 1.57 | 1.57 | 1.61 | 1.6 | 1.62 | 1.59 |
| Cys | UGC | 0.44 | 0.42 | 0.45 | 0.4 | 0.42 | 0.42 | 0.45 | 0.45 | 0.43 | 0.43 | 0.44 | 0.5 | 0.46 | 0.46 | 0.54 |
| Cys | UGU | 1.56 | 1.58 | 1.55 | 1.6 | 1.58 | 1.58 | 1.55 | 1.55 | 1.57 | 1.57 | 1.56 | 1.5 | 1.54 | 1.54 | 1.46 |
| Gln | CAA | 1.59 | 1.6 | 1.58 | 1.6 | 1.59 | 1.59 | 1.58 | 1.58 | 1.6 | 1.59 | 1.6 | 1.53 | 1.55 | 1.53 | 1.49 |
| Gln | CAG | 0.41 | 0.4 | 0.42 | 0.4 | 0.41 | 0.41 | 0.42 | 0.42 | 0.4 | 0.41 | 0.4 | 0.47 | 0.45 | 0.47 | 0.51 |
| Glu | GAA | 1.56 | 1.56 | 1.57 | 1.6 | 1.55 | 1.55 | 1.54 | 1.55 | 1.56 | 1.52 | 1.52 | 1.51 | 1.47 | 1.48 | 1.47 |
| Glu | GAG | 0.44 | 0.44 | 0.43 | 0.5 | 0.45 | 0.45 | 0.46 | 0.45 | 0.44 | 0.48 | 0.48 | 0.49 | 0.53 | 0.52 | 0.53 |
| Gly | GGA | 1.56 | 1.6 | 1.59 | 1.6 | 1.59 | 1.59 | 1.59 | 1.61 | 1.59 | 1.64 | 1.64 | 1.65 | 1.6 | 1.64 | 1.6 |
| Gly | GGC | 0.45 | 0.44 | 0.44 | 0.5 | 0.45 | 0.45 | 0.45 | 0.46 | 0.45 | 0.45 | 0.44 | 0.41 | 0.42 | 0.4 | 0.43 |
| Gly | GGG | 0.64 | 0.62 | 0.63 | 0.6 | 0.63 | 0.63 | 0.64 | 0.59 | 0.61 | 0.58 | 0.58 | 0.67 | 0.64 | 0.66 | 0.72 |
| Gly | GGU | 1.35 | 1.34 | 1.34 | 1.3 | 1.34 | 1.34 | 1.32 | 1.35 | 1.34 | 1.34 | 1.34 | 1.27 | 1.33 | 1.29 | 1.26 |
| His | CAC | 0.41 | 0.4 | 0.39 | 0.4 | 0.41 | 0.4 | 0.39 | 0.43 | 0.4 | 0.42 | 0.41 | 0.47 | 0.43 | 0.43 | 0.46 |
| His | CAU | 1.59 | 1.6 | 1.61 | 1.6 | 1.59 | 1.6 | 1.61 | 1.57 | 1.6 | 1.58 | 1.59 | 1.53 | 1.57 | 1.57 | 1.54 |
| Ile | AUA | 0.93 | 0.94 | 0.93 | 0.9 | 0.93 | 0.93 | 0.93 | 0.95 | 0.92 | 0.93 | 0.93 | 0.95 | 0.96 | 0.93 | 0.9 |
| Ile | AUC | 0.57 | 0.55 | 0.55 | 0.6 | 0.56 | 0.56 | 0.56 | 0.55 | 0.57 | 0.53 | 0.53 | 0.6 | 0.6 | 0.62 | 0.62 |

**(Continued) Table S8. Statistics on the Relative Synonymous Codon Usage (RSCU) for the chloroplast genomes of *Agapetes* and 12 other species.**

| **Amino Acids** | **Codon** | ***A. malipoensis*** | ***A. guangxiensis*** | ***A. obovata*** | ***V. ashei*** | ***V. angustifolium*** | ***V. corymbosum*** | ***V. japonicum*** | ***V. macrocarpon*** | ***V. oxycoccos*** | ***G. griffithiana*** | ***G. fragrantissima*** | ***C. fargesii*** | ***A. eriantha*** | ***S. monadelpha*** | ***S. melongena*** |
| --- | --- | --- | --- | --- | --- | --- | --- | --- | --- | --- | --- | --- | --- | --- | --- | --- |
| Ile | AUU | 1.5 | 1.51 | 1.51 | 1.5 | 1.51 | 1.51 | 1.51 | 1.5 | 1.51 | 1.53 | 1.54 | 1.45 | 1.44 | 1.45 | 1.48 |
| Leu | CUA | 0.82 | 0.79 | 0.8 | 0.8 | 0.8 | 0.8 | 0.8 | 0.74 | 0.78 | 0.78 | 0.79 | 0.8 | 0.82 | 0.82 | 0.82 |
| Leu | CUC | 0.32 | 0.32 | 0.32 | 0.3 | 0.33 | 0.33 | 0.36 | 0.35 | 0.32 | 0.35 | 0.34 | 0.42 | 0.42 | 0.42 | 0.41 |
| Leu | CUG | 0.32 | 0.32 | 0.32 | 0.3 | 0.32 | 0.32 | 0.32 | 0.36 | 0.32 | 0.31 | 0.31 | 0.41 | 0.39 | 0.36 | 0.4 |
| Leu | CUU | 1.25 | 1.23 | 1.24 | 1.2 | 1.23 | 1.24 | 1.24 | 1.2 | 1.23 | 1.23 | 1.24 | 1.27 | 1.26 | 1.26 | 1.31 |
| Leu | UUA | 2.17 | 2.23 | 2.22 | 2.2 | 2.2 | 2.2 | 2.19 | 2.16 | 2.23 | 2.15 | 2.16 | 1.86 | 1.87 | 1.9 | 1.82 |
| Leu | UUG | 1.11 | 1.1 | 1.1 | 1.1 | 1.11 | 1.11 | 1.1 | 1.2 | 1.12 | 1.17 | 1.16 | 1.24 | 1.24 | 1.24 | 1.24 |
| Lys | AAA | 1.57 | 1.58 | 1.57 | 1.6 | 1.56 | 1.56 | 1.55 | 1.58 | 1.57 | 1.51 | 1.51 | 1.51 | 1.46 | 1.46 | 1.49 |
| Lys | AAG | 0.43 | 0.42 | 0.43 | 0.4 | 0.44 | 0.44 | 0.45 | 0.42 | 0.43 | 0.49 | 0.49 | 0.49 | 0.54 | 0.54 | 0.51 |
| Phe | UUC | 0.62 | 0.61 | 0.61 | 0.6 | 0.62 | 0.62 | 0.62 | 0.62 | 0.62 | 0.61 | 0.61 | 0.72 | 0.71 | 0.75 | 0.71 |
| Phe | UUU | 1.38 | 1.39 | 1.39 | 1.4 | 1.38 | 1.38 | 1.38 | 1.38 | 1.38 | 1.39 | 1.39 | 1.28 | 1.29 | 1.25 | 1.29 |
| Pro | CCA | 1.23 | 1.21 | 1.2 | 1.2 | 1.22 | 1.22 | 1.22 | 1.2 | 1.24 | 1.2 | 1.2 | 1.15 | 1.17 | 1.18 | 1.18 |
| Pro | CCC | 0.71 | 0.69 | 0.72 | 0.7 | 0.71 | 0.71 | 0.73 | 0.77 | 0.73 | 0.76 | 0.78 | 0.72 | 0.69 | 0.7 | 0.76 |
| Pro | CCG | 0.44 | 0.47 | 0.45 | 0.5 | 0.45 | 0.45 | 0.47 | 0.43 | 0.44 | 0.47 | 0.46 | 0.55 | 0.54 | 0.49 | 0.55 |
| Pro | CCU | 1.61 | 1.63 | 1.63 | 1.6 | 1.62 | 1.62 | 1.59 | 1.6 | 1.6 | 1.57 | 1.57 | 1.58 | 1.6 | 1.63 | 1.52 |
| Ser | AGC | 0.4 | 0.36 | 0.4 | 0.4 | 0.41 | 0.41 | 0.41 | 0.45 | 0.39 | 0.34 | 0.35 | 0.35 | 0.39 | 0.34 | 0.35 |
| Ser | AGU | 1.23 | 1.26 | 1.23 | 1.2 | 1.22 | 1.22 | 1.21 | 1.22 | 1.23 | 1.26 | 1.25 | 1.2 | 1.16 | 1.22 | 1.19 |
| Ser | UCA | 1.09 | 1.13 | 1.12 | 1.1 | 1.1 | 1.1 | 1.12 | 1.05 | 1.11 | 1.11 | 1.12 | 1.15 | 1.14 | 1.15 | 1.22 |
| Ser | UCC | 0.84 | 0.87 | 0.88 | 0.9 | 0.84 | 0.84 | 0.87 | 0.85 | 0.88 | 0.85 | 0.84 | 0.95 | 0.96 | 0.97 | 0.97 |
| Ser | UCG | 0.52 | 0.52 | 0.52 | 0.6 | 0.53 | 0.53 | 0.56 | 0.55 | 0.52 | 0.59 | 0.59 | 0.57 | 0.56 | 0.54 | 0.59 |
| Ser | UCU | 1.92 | 1.86 | 1.86 | 1.9 | 1.9 | 1.9 | 1.84 | 1.88 | 1.87 | 1.86 | 1.86 | 1.79 | 1.79 | 1.78 | 1.68 |
| Thr | ACA | 1.19 | 1.23 | 1.2 | 1.2 | 1.19 | 1.19 | 1.22 | 1.2 | 1.21 | 1.23 | 1.23 | 1.25 | 1.24 | 1.21 | 1.21 |
| Thr | ACC | 0.75 | 0.73 | 0.76 | 0.8 | 0.77 | 0.77 | 0.77 | 0.74 | 0.74 | 0.76 | 0.76 | 0.74 | 0.72 | 0.72 | 0.77 |
| Thr | ACG | 0.38 | 0.37 | 0.39 | 0.4 | 0.38 | 0.38 | 0.37 | 0.4 | 0.39 | 0.37 | 0.37 | 0.39 | 0.38 | 0.41 | 0.44 |
| Thr | ACU | 1.68 | 1.67 | 1.66 | 1.7 | 1.67 | 1.67 | 1.65 | 1.65 | 1.66 | 1.64 | 1.64 | 1.61 | 1.66 | 1.66 | 1.58 |
| Tyr | UAC | 0.35 | 0.35 | 0.35 | 0.4 | 0.36 | 0.35 | 0.35 | 0.33 | 0.36 | 0.34 | 0.35 | 0.38 | 0.39 | 0.39 | 0.39 |
| Tyr | UAU | 1.65 | 1.65 | 1.65 | 1.7 | 1.64 | 1.65 | 1.65 | 1.67 | 1.64 | 1.66 | 1.65 | 1.62 | 1.61 | 1.61 | 1.61 |

**(Continued) Table S8. Statistics on the Relative Synonymous Codon Usage (RSCU) for the chloroplast genomes of Agapetes and 12 other species.**

| **Amino Acids** | **Codon** | | **A. malipoensis** | | **A. guangxiensis** | | **A. obovata** | | **V. ashei** | | **V. angustifolium** | | **V. corymbosum** | | **V. japonicum** | | **V. macrocarpon** | | **V. oxycoccos** | | **G. griffithiana** | | **G. fragrantissima** | | **C. fargesii** | | **A. eriantha** | | **S. monadelpha** | | **S. melongena** | |
| --- | --- | --- | --- | --- | --- | --- | --- | --- | --- | --- | --- | --- | --- | --- | --- | --- | --- | --- | --- | --- | --- | --- | --- | --- | --- | --- | --- | --- | --- | --- | --- | --- |
| Val | | GUA | | 1.45 | | 1.44 | | 1.47 | | 1.5 | | 1.44 | | 1.44 | | 1.44 | | 1.44 | | 1.46 | | 1.43 | | 1.43 | | 1.52 | | 1.48 | | 1.5 | | 1.48 |
| Val | | GUC | | 0.48 | | 0.5 | | 0.5 | | 0.5 | | 0.52 | | 0.52 | | 0.52 | | 0.51 | | 0.53 | | 0.58 | | 0.57 | | 0.45 | | 0.44 | | 0.46 | | 0.5 |
| Val | | GUG | | 0.48 | | 0.49 | | 0.48 | | 0.5 | | 0.49 | | 0.49 | | 0.5 | | 0.51 | | 0.49 | | 0.48 | | 0.47 | | 0.57 | | 0.58 | | 0.56 | | 0.55 |
| Val | | GUU | | 1.58 | | 1.57 | | 1.55 | | 1.6 | | 1.55 | | 1.55 | | 1.55 | | 1.54 | | 1.53 | | 1.52 | | 1.53 | | 1.46 | | 1.5 | | 1.48 | | 1.46 |

**Table S9. Morphological Characteristics of *Agapetes* and *Vaccinium* Species.**

| **Species** | **Calyx lobe** | **Corolla** | **Style** | **Filament** | **Fruit** | **Phenology** | **Pedicel** | **Leaf** | **Reference** |
| --- | --- | --- | --- | --- | --- | --- | --- | --- | --- |
| *A. malipoensi* | Triangular, 3–6 mm, densely glandular setose or glabrous | White, tubular, glandular hairs, ca. 2 cm | Glabrous, truncate | 1.4 cm, densely pubescent | Berry globose | Fl. Oct–Dec, fr. Oct–Jan | Slightly bullate, secondary veins 5–7 pairs | Leaves scattered and subopposite, elliptic or oblong-lanceolate, leathery, glabrous, margin recurved, entire, terete, densely glandular setose | (Conlon, 2015) |
| *A. guangxiensis* | Triangular, 5 mm, hirsute, pubescent | White, pale green, subtubular, slightly 5-angled | None | Ca. 4 mm Pubescent | Berry | Fl. Sep | Fine veins inconspicuous | Leaves dense, ovate, leathery, glabrous, setose, terete, densely glandular setose | (Conlon, 2015) |
| *A. obovata* | Triangular lanceolate, 0.8–1 cm, glabrous | Deep red, campanulate | Glabrous, truncate | Ca. 1.5 mm, pilose | Berry globose | Fl. Mar | Secondary veins inconspicuous | Leaves crowded, obovate-spatulate, leathery, glabrous, margin recurved, entire, terete, glabrous or spreading hispid | (Conlon, 2015) |
| *A. hongheensis* | Triangular, 7–11 mm, glabrous or sparsely pubescent | Yellowish green, tubular, glabrous on both sides | Glabrous, truncate | flat, 1.2–1.3 cm long, densely pubescent | Berry globose | Fl Sep-Oct. fr in January the next year. | Lateral veins raised, veinlets conspicuous | Leaves spirally alternate, linear or narrowly oblong, leathery, glabrous, margin recurved, entire, slightly angled, glabrous | (Zou et al., 2025) |

**(Continued) Table S9. Morphological Characteristics of *Agapetes* and *Vaccinium* Species.**

| **Species** | **Calyx lobe** | **Corolla** | **Style** | **Filament** | **Fruit** | **Phenology** | **Pedicel** | **Leaf** | **Reference** |
| --- | --- | --- | --- | --- | --- | --- | --- | --- | --- |
| *A. xiana* | Triangular, 3.5–4.2 cm, glabrous | Yellowish green, tubular, glabrous | Glabrous, capitate | Ca. 3 mm long, glabrous | None | None | Impressed midvein, secondary veins 6–8 pairs | Scattered, oblong-lanceolate, leathery, glabrous, margin entire, slightly angled, lenticellate | (Tong, 2016) |
| *A. reflexiloba* | Triangular, 1.5–3 cm, obconical, glabrous | Light brownish yellow, glabrous | Ca. 2.3 cm | flat, glabrous | None | None | Secondary veins inconspicuous | Leaves alternate, lanceolate, glabrous, terete, glabrous | (Yang et al., 2019) |
| *A. yingjiangensis* | Triangular, 2–4 mm, sparsely hirsute | Greenish white | Slender, ca 1.6 cm long, punctate | Stamens 10, flat, densely pubescent | Berry globose | None | secondary veins inconspicuous | Leaves scattered, leathery, obovate to elliptic, with entire margin, slightly angled, densely hirsute | (Tong et al., 2019) |
| *A. brevipedicellata* | Triangular or ovate-Triangular, stout, fleshy, 0.8–1.0 cm, glabrous | Pale pink | Glabrous, ca. 1.6 cm, punctate | Flat, Subglabrous | None | Fl. Apr–Jun | Inconspicuous secondary veins | Leaves dense, ovate, coriaceous, glabrous, margins entire, slender, glabrous | (Zhou et al., 2017) |
| *A. oligodonta* | Lobes 5, ovate-triangular, 2–3 mm, glabrous | White or pale pink, urceolate | 8.0–8.5 mm long, glabrous, truncate | Slightly inflexed, 3.5–3.7 mm | 8.0–8.5 mm, glabrous | Fl. Apr–Jun.fr in January the next year. | Arc-ascending secondary veins | Leaves pseudo verticillate, obovate, glabrous, margins entire, ridged, densely covered with setae | (Tong et al., 2022) |

**(Continued) Table S9. Morphological Characteristics of *Agapetes* and *Vaccinium* Species.**

| **Species** | **Calyx lobe** | **Corolla** | **Style** | **Filament** | **Fruit** | **Phenology** | **Pedicel** | **Leaf** | **Reference** |
| --- | --- | --- | --- | --- | --- | --- | --- | --- | --- |
| *A. lihengiana* | Lobes 5, triangular-lanceolate, 0.5–1.0 cm, densely puberulous | White | 3–18 mm, stigma punctate | Parallel, flattened, Dense white pilosity | None | Fl. Jun–Jul, fr. Nov–Dec | Secondary veins 2–4 pairs | Leaves spirally alternate around stem, ovate, elliptic-ovate to suborbicular, terete, slender, covered with setae | (Yang et al., 2023) |
| *V. japonicum* | Triangular, divided nearly to base | White, pinkish, glabrous | None | 1–2 mm, often densely villous | Berry | Fl. Jun–Jul, Fr. Aug–Oct | 5–8 mm, slender, glabrous | Leaves scattered, ovate or ovate-lanceolate, papery, abaxially subglabrous, margin plane, Fine veins raised abaxially, impressed adaxially | (Mingyuan et al., 2005) |
| *V. oxycoccos* | Reflexed, oblong, suborbicular, ca. 0.5 mm | Pinkish, glabrous | Truncate stigma | Ca. 1.5 mm, ciliolate | Berry | Fl. Jun–Jul, Fr. Jul–Aug | Filiform, 1–2(–3) cm, pubescent | Leaves dense, oblong, leathery, glabrous, margin strongly revolute, entire, secondary veins inconspicuous on both surfaces | (Mingyuan et al., 2005) |
| *V. dehongense* | Recurved, triangular lobes, glabrous | Greenish-yellow, urceolate, 5-angled, glabrous on both surfaces, | 4.5–5.5 mm, capitate | Flat, glabrous | None | None | 6–7.5 mm, glabrous | Pseudo verticillate, firmly papery, oblong-lanceolate, margin plane, fine veins raised on both surfaces. | (Tong et al., 2024) |

**(Continued) Table S9. Morphological Characteristics of *Agapetes* and *Vaccinium* Species.**

| **Species** | **Calyx lobe** | **Corolla** | **Style** | **Filament** | **Fruit** | **Phenology** | **Pedicel** | **Leaf** | **Reference** |
| --- | --- | --- | --- | --- | --- | --- | --- | --- | --- |
| *V. exiguum* | Triangular, 0.7–1.0 mm long, glabrous | Red or pink, broadly | 4.0–5.0 mm, glabrous | Straight, 1.9–2.0 mm | None | None | Nodding, white-puberulent | Leaves densely, elliptic or oblong, glabrous, secondary veins inconspicuous | (Tamayo et al., 2021) |
| *V. motuoense* | Ovate-triangular, lobes 5, margin ciliate | White or red, 5-lobed | Cylindrical, glabrous, punctate | Flat, slightly S-shaped, 1.5–2.0 mm | Berry, globose | None | 2.5–4.5 mm, nearly glabrous | Leaves alternate, oblong, leathery, entire, short cross-veins between the secondaries prominent | (Tong et al., 2021) |
| *V. usneoides* | Ovate-triangular, lobes 5, ca. 2 × 1.5 mm, margin ciliate | Red, urceolate-campanulate | Cylindrical,2.7–3 mm, glabrous, truncate | Flat, 1–1.2 mm, margin pilose | Berry, globose | None | Greenish, short, 0.5–1 mm long, densely pubescent | Leaves alternate, narrowly ovate, densely pubescent, thickly leathery, margin entire, secondary veins inconspicuous | (Yongjie et al., 2023) |
| *V. napoense* | Ovate-triangular, lobes 5, densely villous abaxially, glabrous adaxially | Yellow-green, urceolate | Glabrous, ca 6.0 mm, stigma punctate | Flat, slightly incurved, ca 2.0 mm, densely villous | None | Fl. Mar–Apr | 7–8 mm, sparsely pilose | Leaves densely, obovate or oblanceolate, thickly leathery, entire, revolute, secondary veins inconspicuous | (Tong et al., 2020) |
| *V. ashei* | None | White | None | None | Berry globose | Fl. Mid-Spring, Fr. Early-Mid Summer | None | Glossy, blue-green, 2-5 cm | https://www.gardenia.net/ |

**(Continued) Table S9. Morphological Characteristics of *Agapetes* and *Vaccinium* Species.**

| **Species** | **Calyx lobe** | **Corolla** | **Style** | **Filament** | **Fruit** | **Phenology** | **Pedicel** | **Leaf** | **Reference** |
| --- | --- | --- | --- | --- | --- | --- | --- | --- | --- |
| *V. angustifolium* | None | White, pink-tinged | None | None | Berry globose | Fl. Spring, Fr. Summer | None | Glossy, lustrous, lanceolate leaf shape | https://www.gardenia.net/ |
| *V. corymbosum* | None | flowers are long bell- or urn-shaped white to very light pink | None | None | Berry globose | Fl. Late Spring, Fr. Mid-Summer | None | Ovate, 5 cm long, glossy green | https://www.gardenia.net/ |
| *V. macrocarpon* | None | White or pinkish | None | None | Berry globose | Fl. Late Spring, Fr. Early Fall | None | Ovate, narrowly elliptic, 1–2 cm long | https://www.gardenia.net/ |

Note: None in the table indicates no data found.

**Table S10. Shared and specific Features of *Agapete*s and *Vaccinium* Species**

|  | **Common Characteristics** | ***Agapetes* specific characteristics** | ***Vaccinium* specific characteristics** |
| --- | --- | --- | --- |
| **Calyx lobe** | Mostly triangular, usually 5-lobed; outer surface often glabrous | Calyx large | Calyx short |
| **Corolla** | Corolla typically tubular or urceolate, with color polymorphism, glabrous | Corolla usually large, with diverse coloration | Corolla usually small, predominantly red, white, or pink |
| **Style** | Single, glabrous; stigma often punctate or truncate | Style large | Style short |
| **Filament** | Mostly flattened, pubescent or glabrous; base often slightly dilated | Filaments large | Filaments short |
| **Fruit** | Fruits mostly berry-like and globose | No diagnostic traits | No diagnostic traits |
| **Phenology** | Flowering in spring–summer, fruiting in autumn–winter | Some species flower in winter and fruit the following spring | Flowering in spring–summer, fruiting in autumn–winter |
| **Pedicel** | Both possess elongate pedicels, mostly glabrous | Pedicels large | Pedicels relatively short, some species pubescent |
| **Leaf** | Alternate or subverticillate, mostly leathery, with revolute or entire margins, glabrous. Lateral veins all inconspicuous | Leaf shape mostly oblong or lanceolate. Secondary veins/fine veinlets often conspicuously raised and frequently anastomosing on the adaxial surface. | Leaf shape mostly orbicular or ovate. Leaf venation rather variable. |

**Table S11. GC content of different cp genome regions of *Agapetes* and relatives.**

| **Group** | **Species** | **LSC (%)** | **IRb (%)** | **SSC (%)** | **CP (%)** |
| --- | --- | --- | --- | --- | --- |
| *Agapetes* (AGA) | *A. malipoensis* | 35.73 | 38.63 | 27.89 | 36.67 |
|  | *A. guangxiensis* | 35.52 | 38.36 | 27.32 | 36.47 |
|  | *A. obovata* | 35.75 | 38.38 | 27.87 | 36.65 |
| *Vaccinium* (VAC) | *V. ashei* | 35.91 | 38.58 | 29.33 | 36.81 |
|  | *V. angustifolium* | 35.90 | 38.44 | 29.46 | 36.80 |
|  | *V. corymbosum* | 35.89 | 38.37 | 29.37 | 36.80 |
|  | *V. japonicum* | 35.72 | 38.35 | 29.33 | 36.74 |
|  | *V. macrocarpon* | 35.77 | 38.73 | 28.72 | 36.80 |
|  | *V. oxycoccos* | 35.81 | 38.49 | 28.31 | 36.74 |
| Other (OUT) | *G. griffithiana* | 35.64 | 38.70 | 27.81 | 36.63 |
|  | *G. fragrantissima* | 35.60 | 38.74 | 28.01 | 36.00 |
|  | *C. fargesii* | 35.38 | 42.97 | 30.73 | 37.34 |
|  | *A. eriantha* | 35.43 | 43.34 | 31.09 | 37.19 |
|  | *S. monadelpha* | 35.27 | 42.87 | 30.54 | 37.25 |
|  | *S. melongena* | 35.79 | 43.05 | 31.94 | 37.71 |

**Table S12. Chloroplast genomes composition of *Agapetes* and relatives**

| **Group** | **Species** | **Coding (%)** | **Non-Coding (%)** | **PCG (count)** |
| --- | --- | --- | --- | --- |
| *Agapetes* (AGA) | *A. malipoensis* | 50.15 | 49.85 | 85 |
|  | *A. guangxiensis* | 50.38 | 49.62 | 85 |
|  | *A. obovata* | 49.16 | 50.84 | 85 |
| *Vaccinium* (VAC) | *V. ashei* | 46.79 | 53.21 | 85 |
|  | *V. angustifolium* | 46.47 | 53.53 | 85 |
|  | *V. corymbosum* | 45.35 | 54.65 | 85 |
|  | *V. japonicum* | 46.05 | 53.95 | 88 |
|  | *V. macrocarpon* | 47.81 | 52.19 | 85 |
|  | *V. oxycoccos* | 47.92 | 52.08 | 84 |
| Other (OUT) | *G. griffithiana* | 50.99 | 49.01 | 90 |
|  | *G. fragrantissima* | 51.58 | 48.42 | 92 |
|  | *C. fargesii* | 67.20 | 32.80 | 87 |
|  | *A. eriantha* | 62.07 | 37.93 | 84 |
|  | *S. monadelpha* | 71.46 | 28.54 | 89 |
|  | *S. melongena* | 85.64 | 14.36 | 89 |

**Table S13.** **Counts of different types of simple sequence repeats (SSR) for chloroplast genomes of *Agapetes* and relatives.**

| **SSR type** | **Repeta unit** | ***A. malipoensis*** | ***A. guangxiensis*** | ***A. obovata*** | ***V. ashei*** | ***V. angustifolium*** | ***V. corymbosum*** | ***V. japonicum*** | ***V. macrocarpon*** | ***V. oxycoccos*** | ***G. griffithiana*** | ***G. fragrantissima*** | ***C. fargesii*** | ***A. eriantha*** | ***S. monadelpha*** | ***S. melongena*** |
| --- | --- | --- | --- | --- | --- | --- | --- | --- | --- | --- | --- | --- | --- | --- | --- | --- |
| Mono | A/T | 34 | 43 | 28 | 31 | 42 | 46 | 27 | 25 | 34 | 36 | 40 | 18 | 36 | 43 | 44 |
| Mono | C/G | 0 | 0 | 1 | 0 | 1 | 1 | 0 | 1 | 3 | 0 | 0 | 0 | 4 | 0 | 0 |
| Count |  | 34 | 43 | 29 | 31 | 43 | 47 | 27 | 26 | 37 | 36 | 40 | 18 | 40 | 43 | 44 |
| Di | AT/AT | 10 | 18 | 8 | 17 | 19 | 21 | 10 | 9 | 9 | 8 | 9 | 5 | 6 | 7 | 7 |
| Tri | AAG/CTT | 2 | 2 | 6 | 3 | 3 | 3 | 2 | 4 | 4 | 5 | 6 | 1 | 1 | 1 | 1 |
| Tri | ACT/AGT | 0 | 4 | 0 | 0 | 0 | 0 | 0 | 0 | 0 | 0 | 0 | 0 | 0 | 0 | 0 |
| Tri | AAT/ATT | 4 | 4 | 2 | 3 | 3 | 3 | 2 | 2 | 2 | 3 | 2 | 2 | 6 | 3 | 5 |
| Tri | ATC/ATG | 0 | 0 | 0 | 0 | 0 | 0 | 17 | 0 | 0 | 2 | 2 | 0 | 0 | 0 | 0 |
| Count |  | 6 | 10 | 8 | 6 | 6 | 6 | 21 | 6 | 6 | 10 | 10 | 3 | 7 | 4 | 6 |
| Tetra | AACC/GGTT | 0 | 6 | 6 | 0 | 0 | 0 | 0 | 2 | 0 | 0 | 0 | 0 | 0 | 0 | 0 |
| Tetra | AAAG/CTTT | 9 | 8 | 14 | 6 | 6 | 12 | 0 | 10 | 8 | 6 | 6 | 2 | 3 | 3 | 0 |
| Tetra | AAAC/GTTT | 0 | 0 | 0 | 0 | 0 | 0 | 0 | 0 | 0 | 1 | 1 | 0 | 0 | 0 | 3 |
| Tetra | AAAT/ATTT | 10 | 4 | 4 | 4 | 6 | 6 | 4 | 10 | 8 | 2 | 3 | 2 | 3 | 4 | 3 |
| Tetra | AAGG/CCTT | 0 | 0 | 0 | 0 | 0 | 0 | 0 | 0 | 0 | 1 | 0 | 0 | 0 | 0 | 0 |
| Tetra | AATC/ATTG | 2 | 2 | 2 | 2 | 2 | 2 | 2 | 2 | 0 | 3 | 3 | 1 | 1 | 1 | 0 |
| Tetra | AATG/ATTC | 1 | 0 | 0 | 0 | 0 | 0 | 0 | 0 | 0 | 1 | 0 | 0 | 0 | 0 | 0 |
| Tetra | ATCC/ATGG | 1 | 2 | 1 | 1 | 1 | 1 | 0 | 1 | 1 | 1 | 1 | 0 | 0 | 0 | 0 |
| Tetra | ACAG/CTGT | 0 | 0 | 0 | 0 | 0 | 0 | 0 | 0 | 0 | 0 | 0 | 0 | 0 | 1 | 0 |
| Tetra | AGAT/ATCT | 0 | 0 | 0 | 0 | 0 | 0 | 0 | 0 | 0 | 0 | 0 | 0 | 0 | 3 | 2 |
| Tetra | AATT/AATT | 3 | 3 | 7 | 3 | 3 | 3 | 3 | 2 | 3 | 4 | 0 | 0 | 0 | 0 | 0 |
| Count |  | 26 | 25 | 34 | 16 | 18 | 24 | 9 | 27 | 20 | 19 | 14 | 5 | 7 | 12 | 8 |
| Penta | AAAAC/GTTTT | 0 | 0 | 0 | 0 | 0 | 0 | 0 | 0 | 0 | 0 | 0 | 1 | 0 | 0 | 0 |
| Penta | AAAAG/CTTTT | 0 | 0 | 0 | 0 | 0 | 0 | 0 | 0 | 0 | 0 | 0 | 0 | 2 | 0 | 0 |
| Penta | AAATT/AATTT | 0 | 1 | 0 | 0 | 0 | 0 | 0 | 0 | 0 | 0 | 0 | 0 | 1 | 0 | 0 |
| Penta | AAACT/AGTTT | 0 | 0 | 0 | 0 | 0 | 0 | 0 | 0 | 0 | 0 | 0 | 0 | 0 | 1 | 0 |
| Penta | AATGC/ATTGC | 1 | 0 | 0 | 0 | 0 | 0 | 0 | 0 | 0 | 0 | 0 | 0 | 0 | 0 | 0 |
| Penta | ACATG/ATGTC | 0 | 0 | 0 | 0 | 0 | 0 | 0 | 0 | 4 | 0 | 0 | 0 | 0 | 0 | 0 |
| Penta | AATAG/ATTCT | 0 | 0 | 0 | 0 | 0 | 0 | 0 | 10 | 0 | 0 | 0 | 0 | 0 | 0 | 0 |
| Penta | AATTC/AATTG | 0 | 0 | 0 | 0 | 0 | 0 | 0 | 0 | 0 | 0 | 0 | 0 | 0 | 0 | 1 |
| Count |  | 1 | 1 | 0 | 0 | 0 | 0 | 0 | 10 | 4 | 0 | 0 | 1 | 3 | 1 | 1 |
| Hexa | AAGGGT/ACCCTT | 0 | 0 | 0 | 0 | 0 | 0 | 0 | 0 | 0 | 0 | 0 | 0 | 0 | 0 | 0 |
| Hexa | AAAAAG/CTTTTT | 1 | 1 | 1 | 1 | 1 | 1 | 0 | 1 | 1 | 1 | 0 | 0 | 0 | 0 | 0 |
| Hexa | AATAGG/ATTCCT | 4 | 2 | 8 | 2 | 2 | 8 | 10 | 6 | 4 | 0 | 0 | 0 | 0 | 0 | 0 |
| Hexa | ACTCAT/AGTATG | 1 | 0 | 0 | 0 | 0 | 0 | 0 | 0 | 0 | 0 | 0 | 0 | 0 | 0 | 0 |
| Hexa | AATGGG/ATTCCC | 0 | 0 | 0 | 0 | 0 | 0 | 0 | 0 | 0 | 0 | 0 | 0 | 0 | 0 | 0 |
| Hexa | AATAGT/ACTATT | 0 | 0 | 2 | 0 | 0 | 0 | 0 | 0 | 0 | 0 | 0 | 1 | 0 | 0 | 0 |
| Count |  | 6 | 3 | 11 | 3 | 3 | 9 | 10 | 7 | 5 | 1 | 0 | 1 | 0 | 0 | 0 |
| **Total** |  | 83 | 100 | 90 | 73 | 89 | 107 | 77 | 85 | 81 | 74 | 73 | 33 | 63 | 67 | 66 |

**Table S14. Statistics on Dispersed repeat types for the chloroplast genomes of *Agapetes* and 12 other species.**

| Type | Repeta Uint | *A. malipoensis* | *A. guangxiensis* | *A. obovata* | *V. ashei* | *V. angustifolium* | *V. corymbosum* | *V. japonicum* | *V. macrocarpon* | *V. oxycoccos* | *G. griffithiana* | *G. fragrantissima* | *C. fargesii* | *A. eriantha* | *S. monadelpha* | *S. melongena* |
| --- | --- | --- | --- | --- | --- | --- | --- | --- | --- | --- | --- | --- | --- | --- | --- | --- |
| Forward | 30-100 | 534 | 676 | 992 | 753 | 788 | 493 | 1256 | 797 | 1085 | 438 | 397 | 18 | 92 | 20 | 19 |
| Forward | 100-200 | 31 | 45 | 115 | 75 | 172 | 152 | 216 | 91 | 184 | 43 | 34 | 0 | 6 | 0 | 0 |
| Forward | 200-300 | 21 | 15 | 12 | 14 | 22 | 34 | 95 | 12 | 54 | 11 | 6 | 0 | 0 | 0 | 0 |
| Forward | 300-1000 | 5 | 7 | 14 | 16 | 55 | 58 | 125 | 22 | 14 | 9 | 5 | 0 | 0 | 0 | 0 |
| Forward | 1000-10000 | 2 | 8 | 8 | 0 | 1 | 13 | 24 | 4 | 0 | 2 | 3 | 0 | 0 | 0 | 0 |
| Count |  | 593 | 751 | 1141 | 858 | 1038 | 750 | 1716 | 926 | 1337 | 503 | 445 | 18 | 98 | 20 | 19 |
| Palindromic | 30-100 | 483 | 597 | 699 | 708 | 709 | 464 | 1263 | 751 | 1137 | 371 | 325 | 17 | 35 | 22 | 20 |
| Palindromic | 100-200 | 27 | 38 | 51 | 59 | 166 | 151 | 181 | 109 | 162 | 39 | 32 | 0 | 3 | 0 | 0 |
| Palindromic | 200-300 | 19 | 15 | 13 | 13 | 25 | 41 | 85 | 9 | 54 | 10 | 8 | 0 | 0 | 0 | 0 |
| Palindromic | 300-1000 | 4 | 8 | 7 | 18 | 46 | 49 | 135 | 25 | 16 | 11 | 7 | 0 | 1 | 0 | 0 |
| Palindromic | 1000-10000 | 4 | 9 | 11 | 0 | 2 | 17 | 23 | 4 | 1 | 3 | 4 | 0 | 0 | 0 | 0 |
| Palindromic | >10000 | 1 | 1 | 1 | 1 | 1 | 1 | 1 | 1 | 1 | 1 | 1 | 1 | 1 | 2 | 1 |
| Count |  | 538 | 668 | 782 | 799 | 949 | 723 | 1688 | 899 | 1371 | 435 | 377 | 18 | 40 | 24 | 21 |
| Reverse | 30-100 | 28 | 5 | 0 | 2 | 0 | 0 | 1 | 0 | 0 | 4 | 0 | 1 | 6 | 2 | 0 |
| Reverse | 100-200 | 4 | 0 | 0 | 0 | 0 | 0 | 0 | 0 | 0 | 0 | 0 | 0 | 0 | 0 | 0 |
| Count |  | 32 | 5 | 1 | 2 | 0 | 0 | 1 | 0 | 0 | 4 | 0 | 1 | 6 | 2 | 0 |
| complement | 30-100 | 0 | 1 | 1 | 0 | 0 | 0 | 0 | 0 | 0 | 2 | 1 | 0 | 0 | 0 | 0 |
| Total |  | 1163 | 1425 | 1925 | 1659 | 1987 | 1473 | 3405 | 1825 | 2708 | 944 | 823 | 37 | 144 | 46 | 40 |

**Table S15. 219-bp Homologous sequence information**

| **Species** | **Indices** | **Period Size** | **Copy Number** | **Consensus Size** | **Percent Matches** | **Percent Indels** | **Score** |
| --- | --- | --- | --- | --- | --- | --- | --- |
| *A. malipoensis* | 80815--81376 | 219 | 2.6 | 219 | 87 | 1 | 750 |
| *A. guangxiensis* | 80981--81542 | 219 | 2.6 | 219 | 88 | 0 | 784 |
| *A. obovata* | 81676--82236 | 219 | 2.6 | 218 | 86 | 2 | 741 |
| *V. ashei* | 81843--82433 | 219 | 2.7 | 219 | 87 | 2 | 830 |
| *V. angustifolium* | 82594--83184 | 219 | 2.7 | 219 | 87 | 2 | 812 |
| *V. corymbosum* | 81560--82150 | 219 | 2.7 | 219 | 87 | 2 | 812 |
| *V. japonicum* | 80609--81207 | 219 | 2.7 | 219 | 91 | 1 | 916 |
| *V. macrocarpon* | 81071--81661 | 219 | 2.7 | 219 | 87 | 2 | 812 |
| *V. oxycoccos* | 81332--81922 | 219 | 2.7 | 219 | 87 | 2 | 812 |
| *G. griffithiana* | 24743--24525 | 219 |  |  |  |  |  |
| *G. fragrantissima* | 24809--24591 | 219 |  |  |  |  |  |

Note: *Agapetes* and *Vaccinium* sequences are Tandem Reptuer, and the white *Gaultheria* sequence is homologous.

**References**

Conlon, T. (2015). Agapetes D. Don Ex G. Don – Jewels of the East at the Royal Botanic Garden Edinburgh. *Sibbaldia Int. J. Bot. Gard. Hortic.*, 61–82. doi: 10.24823/Sibbaldia.2015.76

Ding, S., Dong, X., Yang, J., Guo, C., Cao, B., Guo, Y., et al. (2021). Complete chloroplast genome of clethra fargesii franch., an original sympetalous plant from central china: Comparative analysis, adaptive evolution, and phylogenetic relationships. *Forests* 12. doi: 10.3390/f12040441

Fahrenkrog, A. M., Matsumoto, G. O., Toth, K., Jokipii-Lukkari, S., Salo, H. M., Häggman, H., et al. (2022). Chloroplast genome assemblies and comparative analyses of commercially important Vaccinium berry crops. *Sci. Rep.* 12, 1–13. doi: 10.1038/s41598-022-25434-5

Kim, Y., Shin, J., Oh, D. R., Kim, A. Y., and Choi, C. (2020). Comparative analysis of complete chloroplast genome sequences and insertion-deletion (Indel) polymorphisms to distinguish five Vaccinium species. *Forests* 11, 1–13. doi: 10.3390/F11090927

Knapp, S., Vorontsova, M. S., and Prohens, J. (2013). Wild Relatives of the Eggplant (Solanum melongena L.: Solanaceae): New Understanding of Species Names in a Complex Group. *PLoS One* 8. doi: 10.1371/journal.pone.0057039

Kron, K. A., Powell, E. A., and Luteyn, J. L. (2002). Phylogenetic relationships within the blueberry tribe (Vaccinieae, Ericaceae) based on sequence data from matK and nuclear ribosomal ITS regions, with comments on the placement of Satyria. *Am. J. Bot.* 89, 327–336. doi: 10.3732/ajb.89.2.327

Li, D. Z., Gao, L. M., Li, H. T., Wang, H., Ge, X. J., Liu, J. Q., et al. (2011). Comparative analysis of a large dataset indicates that internal transcribed spacer (ITS) should be incorporated into the core barcode for seed plants. *Proc. Natl. Acad. Sci. U. S. A.* 108, 19641–19646. doi: 10.1073/pnas.1104551108

Li, Y. R., Xu, Y. L., Du, X. Y., Yang, S. Da, and Lu, L. (2021). Characterization of the complete plastid genome of Gaultheria griffithiana (Ericaceae). *Mitochondrial DNA Part B Resour.* 6, 1575–1577. doi: 10.1080/23802359.2021.1914227

Lin, H. Y., Hao, Y. J., Li, J. H., Fu, C. X., Soltis, P. S., Soltis, D. E., et al. (2019). Phylogenomic conflict resulting from ancient introgression following species diversification in Stewartia s.l. (Theaceae). *Mol. Phylogenet. Evol.* 135, 1–11. doi: 10.1016/j.ympev.2019.02.018

López, K. E. R., Armijos, C. E., Parra, M., and Torres, M. de L. (2023). The First Complete Chloroplast Genome Sequence of Mortiño (Vaccinium floribundum) and Comparative Analyses with Other Vaccinium Species. *Horticulturae* 9. doi: 10.3390/horticulturae9030302

Matuszak, S. (2015). Evolution of mountain plants in the region of the Qinghai-Tibetan Plateau and beyond. 2015.

Mingyuan, F., Ruizheng, F., Mingyou, H., Linzhen, H., Ling-cheng, H., Hanbi, Y., et al. (2005). Flora of China. 242–517.

Qiao, X., Gu, Q., Ye, R., Cai, J., and Zhu, N. (2023). The complete chloroplast genome of Vaccinium oxycoccos (Ericaceae)Yan-Ling, X., Du, X. Y., Yi-Rong, L., and Lu, L. (2021). The complete chloroplast genome of Gaultheria fragrantissima Wall. (Ericaceae) from Yunnan, China, an aromatic medicinal plant in th. *Mitochondrial DNA Part B Resour.* 8, 942–947. doi: 10.1080/23802359.2023.2252943

Tamayo, M. N., Bustamante, R. A. A., and Fritsch, P. W. (2021). Vaccinium exiguum (Ericaceae, Vaccinieae), a new species from the ultramafic summit of Mt. Victoria, Palawan Island, Philippines. *PhytoKeys* 179, 145–154. doi: 10.3897/phytokeys.179.68323

Tang, P., Shen, R., He, R., and Yao, X. (2019). The complete chloroplast genomeDing, Shixiong, Xiang Dong, Jiaxin Yang, Chunce Guo, Binbin Cao, Yuan GuTang, Ping, Ruinan Shen, Ruiwen He, and Xiaohong Yao. 2019. “The Complete Chloroplast GenomeDing, Shixiong, Xiang Dong, Jiaxin Yang, Chunce Guo, Binbin . *Mitochondrial DNA Part B Resour.* 4, 2114–2115. doi: 10.1080/23802359.2019.1623111

Tong, Y.-H., Fritsch, P. W., Tan, Y.-H., Aung, M. M., Yang, B., and Armstrong, K. E. (2022). Novelties in Myanmar Agapetes (Ericaceae) with an updated checklist of species from the country. *Nord. J. Bot.* 2022, e03496. doi: https://doi.org/10.1111/njb.03496

Tong, Y.-H., Huang, Y.-S., Ye, X.-H., Cai, Z.-Y., and Xia, N.-H. (2020). Vaccinium napoense, a new species of V. sect. Conchophyllum (Ericaceae) from Guangxi, China. *Nord. J. Bot.* 38. doi: https://doi.org/10.1111/njb.02773

Tong, Y.-H., Wang, B.-M., and Xia, N.-H. (2019). Agapetes yingjiangensis (Ericaceae), a new epiphytic species of A. ser. Longifiles from Yunnan, China. *Nord. J. Bot.* 37, e02171. doi: https://doi.org/10.1111/njb.02171

Tong, Y.-H., Ye, X.-E., and Ni, J.-B. (2024). Vaccinium dehongense (Ericaceae), a new species of Vaccinium sect. Epigynium from western Yunnan, China. *PhytoKeys* 242, 31–37. doi: https://doi.org/10.3897/phytokeys.242.121623

Tong, Y.-H., Zhao, W.-L., Wang, B.-M., Liu, E.-D., Cai, J., and Guo, Y.-J. (2021). Vaccinium motuoense (Ericaceae), a new species from Xizang, China. *PhytoKeys* 181, 105–111. doi: 10.3897/phytokeys.181.71522

Tong, Y. H. (2016). Agapetes xiana sp. Nov. (Ericaceae) from Xizang, China. *Phytotaxa* 252, 289–292. doi: 10.11646/phytotaxa.252.4.6

Yan-Ling, X., Du, X. Y., Yi-Rong, L., and Lu, L. (2021). The complete chloroplast genome of Gaultheria fragrantissima Wall. (Ericaceae) from Yunnan, China, an aromatic medicinal plant in the wintergreens. *Mitochondrial DNA Part B Resour.* 6, 1761–1762. doi: 10.1080/23802359.2021.1923425

Yang, B. I. N., Ding, H., Zhou, S., Maw, M. Y. A. B., Maung, K. W. I. N., and Tan, Y. (2019). Taxonomic studies on Agapetes in Myanmar I: Agapetes reflexiloba, a new species from Kachin State, and notes on three rediscovered species including two new records for Myanmar. *Phytotaxa* 393, 105–118. doi: 10.11646/phytotaxa.393.2.2

Yang, B., Wang, L. Y., Yue, M. M., Ma, X. Da, Yang, Y. J. W., Ma, J. N., et al. (2023). Agapetes lihengiana (Ericaceae), a new species from Yunnan, China. *Taiwania* 68, 355–358. doi: 10.6165/tai.2023.68.355

Yongjie, G., Zhang, T., Ya, J.-D., Zhang, W., Shen, X.-Y., Han, Z.-D., et al. (2023). Vaccinium usneoides (Ericaceae), a new species from Yunnan, China. *PhytoKeys* 236, 187–195. doi: 10.3897/phytokeys.236.112658

Zhou, S. S., Yang, B., Tong, Y. H., Ding, H. B., Li, R., Maung, K. W., et al. (2017). Agapetes brevipedicellata (Ericaceae), a new species from Putao, Kachin state, Northern Myanmar. *Phytotaxa* 331, 117–123. doi: 10.11646/phytotaxa.331.1.10

Zou, C. Y., Wang, B. M., Huang, Y. S., and Tong, Y. H. (2025). Agapetes hongheensis (Ericaceae), a new species from Yunnan, China. *PhytoKeys* 251, 167–174. doi: 10.3897/phytokeys.251.137015
